# Serum IgA and bactericidal immunity against *Streptococcus suis* serotype 2 is increasing between 2 and 6 weeks of age in a farm with autogenous bacterin vaccination pre-farrowing, while specific maternal IgG is decreasing

**DOI:** 10.1186/s40813-025-00485-y

**Published:** 2026-01-14

**Authors:** Theresa Middendorf, Josepha Hallbauer, Matthias Horn, Silke Lehnert, Karoline Rieckmann, Alexander Maas, Wieland Schrödl, Christoph G. Baums

**Affiliations:** 1 Institute of Bacteriology and Mycology, Centre for Infectious Diseases, Faculty of Veterinary Medicine, Leipzig University, An den Tierkliniken 29 04103 Leipzig, Germany; 2 Vivet Schweinegesundheit GmbH, Geseke, Germany; 3 Institute for Medical Informatics, Statistics and Epidemiology (IMISE), Faculty of Medicine, University of Leipzig, 04107 Leipzig, Germany

**Keywords:** Colostrum, IgG, IgA, Maternally-derived antibodies, Bactericidal assay

## Abstract

**Background:**

Neonatal piglets take up maternal IgG and IgA antibodies via colostrum. *Streptococcus suis* (*S. suis*) is a major porcine pathogen that may cause invasive infections in the first ten weeks of life, leading to septicemia, polyarthritis, and meningitis. Preparturient dam vaccination with autogenous *S. suis* vaccines is common in the field. Vaccination with *S. suis* bacterins including water-in-oil adjuvants pre-farrowing elicits increased levels of *S. suis* specific serum IgG antibodies in suckling piglets. However, the influence of various factors associated with colostrum uptake on *S. suis* specific immunity in piglets has not been investigated thoroughly. This field study was designed to investigate the role of colostrum uptake on levels of IgG and IgA binding to *S. suis* and how these specific IgG and IgA levels are associated with bactericidal immunity during the nursery phase.

**Results:**

Levels of serum IgG and IgA antibodies binding to the homologous *S. suis* serotype (*cps*) 2 strain in 2-week-old piglets correlated significantly with respective levels in colostrum. The quantity of individual colostrum uptake in the first 24 h of life, ranging from 250 g to 733 g in the investigated piglets, showed a correlation with *S. suis* specific IgA but no correlation with IgG levels in 2-week-old piglets. Levels of specific serum IgG declined significantly in the following 8 weeks of life whereas levels of serum IgA binding to *S. suis cps*2 increased prominently. Whereas bactericidal immunity in porcine blood against *S. suis cps*2 was rising in most litters between the 2nd and 6th week of life, increased streptococcal survival factors were observed in single litters in the 6th week. Bactericidal immunity at 2, 6 and 10 weeks of age was not associated with colostrum uptake, body weight at birth, the level of specific IgG or IgA in colostrum or the level of specific IgG in serum of the piglets but with the level of serum IgA binding to *S. suis cps*2.

**Conclusions:**

The levels of *S. suis*-specific serum IgG and IgA in 2-week-old suckling piglets in this herd with autogenous bacterin vaccination before farrowing are mainly determined by the respective levels in colostrum. In the following 8 weeks of life, specific serum IgG declines further, whereas specific serum IgA increases prominently and is associated with increased bactericidal immunity in the blood. Our results indicate that an IgG-independent mechanism plays a crucial role in bactericidal immunity after weaning in this herd.

**Supplementary Information:**

The online version contains supplementary material available at 10.1186/s40813-025-00485-y.

## Background

Piglets receive maternal antibodies only through colostrum uptake because the epitheliochorial placentation type does not allow transfer of antibodies. Maternal antibodies in colostrum are mainly IgG, followed by IgA and IgM. In milk, IgA is the dominant immunoglobulin. All IgG in colostrum comes from the sow’s bloodstream. In contrast, IgA and IgM are also produced locally by plasma cells in the mammary gland and secreted directly into the alveoli [1, 2].

*Streptococcus suis* (*S. suis*) causes diseases in suckling, weaning and growing piglets. Arthritis, meningitis, septicemia and endocarditis are common pathological findings of invasive *S. suis* infection. The *S. suis* population includes a broad spectrum of strains ranging from commensals of very low virulence to highly virulent strains causing major herd problems [3]. Serotype (*cps*) 2 strains of clonal complex (CC) 1 are known to be highly virulent [4, 5]. European *cps*2 strains of CC1 generally express the virulence markers suilysin (gene *sly*), muramidase-released protein (MRP, gene *mrp*) and extracellular factor (EF, gene *epf*) [6, 7]. Though *S. suis* strains of lower virulence dominate as colonizers on mucosal surfaces, healthy carriers of highly virulent strains such as *mrp* + *epf* + *sly* + *cps*2 + are generally thought to play an essential epidemiological role, e.g. in the initial transmission from a healthy carrier dam to at least one of its piglets [8].

Preparturient dam vaccination with autogenous bacterins is a common measure in porcine health management in Europe because no licensed, cross-protective vaccine is available [9]. Applying a monovalent *S. suis* cps2 bacterin before farrowing was associated with protection against homologous challenges in 6-week-old but not 8-week-old littermates [10]. Vaccinating dams with bacterins before farrowing is known to elicit increased specific IgG levels in their litters [11, 12]. However, no *S. suis* vaccination protocol has demonstrated increased specific IgA levels so far.

Serum IgA is distinct from mucosal IgA because it lacks the secretory component. In pigs, mucosal and serum IgA consists predominantly of dimers. Colonisation of the intestinal tract of neonatal piglets with bacteria promotes proliferation of IgA + B cells in the ileal Peyer’s Patches, which are likely also involved in generating serum IgA [13]. Myeloid cells, such as monocytes and neutrophilic granulocytes, express the specific IgA receptor FcαRI [14, 15]. In humans, binding of monomeric IgA to FcαRI has been shown to lead to secretion of pro-inflammatory cytokines, phagocytosis, degranulation, and formation of neutrophil extracellular traps (NETs) [16, 17]. A putative role of serum IgA in control of *S. suis* bacteremia in pigs has not been investigated.

This prospective study was conducted in a farrow-to-finish herd that practices vaccination with an autogenous *S. suis* bacterin pre-farrowing. We asked how various factors such as parity, litter position, quantity of colostrum uptake and specific antibody levels in colostrum influence the level of maternally-derived IgG and IgA antibodies against the virulent *S. suis cps*2 strain included in the vaccine and how these antibody levels change until the 10th week of life. A further important objective of this study was to identify parameters that have an effect on killing of *S. suis cps*2 in the blood of piglets.

## Materials and methods

### History and status of the herd

All samples investigated in this study were drawn in a farm located in North Rhine-Westphalia in Germany. This farm of 450 sows with a share of 50% Large White and 50% Landrace F1 sows conducted integration of gilts every 3 weeks. The herd was managed with a 3-week rhythm. Dams and piglets were vaccinated against PRRSV infection, and the herd was considered PRRS stable because the PRRS field virus had not been detected. The herd was free of clinical signs of atrophic rhinitis and mange but had experienced numerous influenza outbreaks. Before introduction, gilts were clinically monitored during an 8-week quarantine and vaccinated against infection with PCV2, SIV, PRRSV, *Rotavirus*,* Escherichia coli*,* Erysipelothrix rhusiopathiae*,* Porcine Parvovirus*, and *Mycoplasma hyopneumoniae*. Furthermore, an autogenous vaccine containing *Actinobacillus pleuropneumoniae*, *Clostridium perfringens* type A, *Escherichia coli*, *Glaesserella parasuis*, *Staphylococcus hyicus*, *Streptococcus dysgalactiae* ssp. *equisimilis* and *Streptococcus suis* (see below) was applied.

### Design of the study

**Fig. 1:**
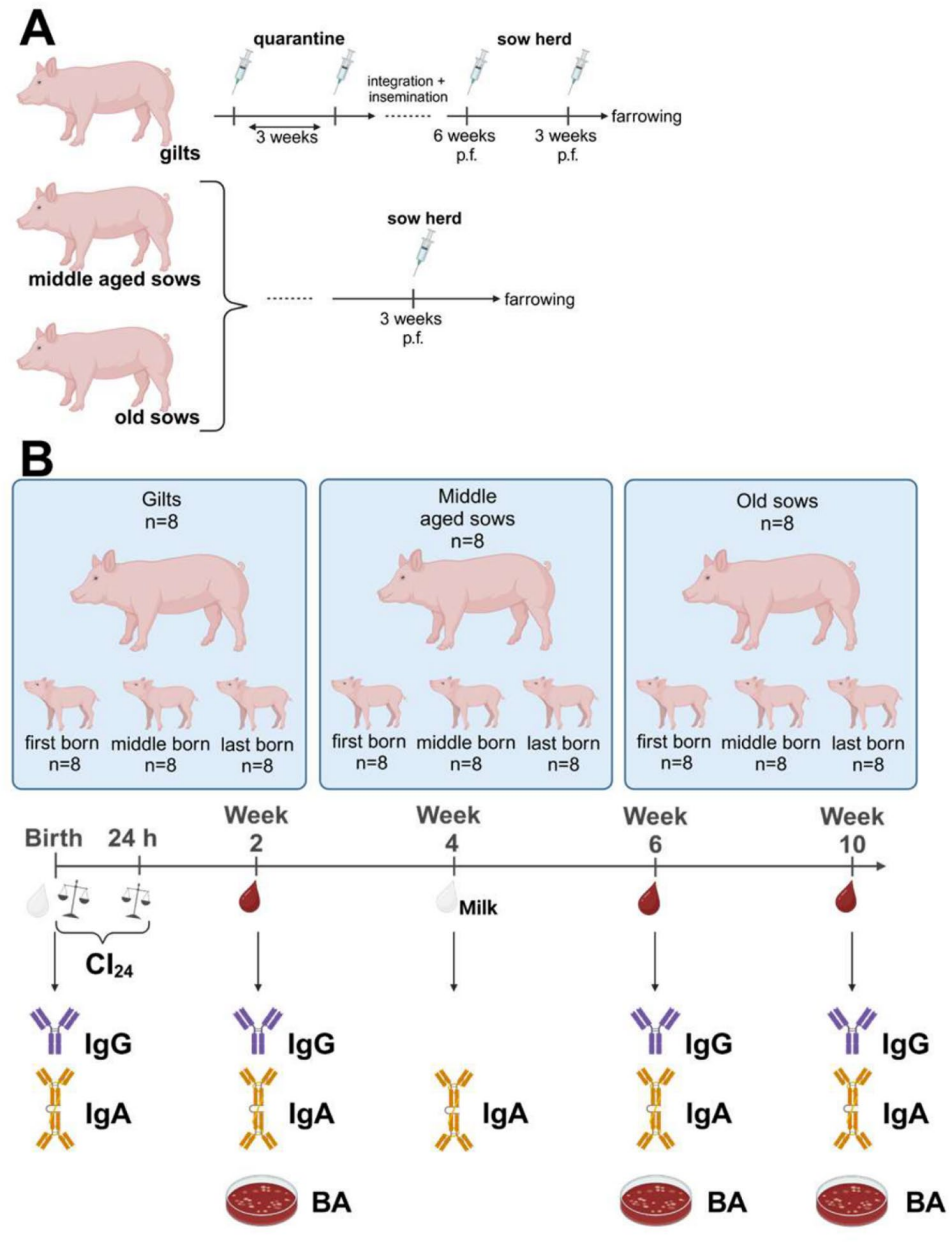
Schedule for vaccination of dams with an autogenous *S. suis* bacterin in the selected herd (**A**) and design, sampling and read out parameters in this longitudinal field study (**B**). The depicted vaccination schedule was established in this herd independently of this field trial and included a boost vaccination 3 weeks pre-farrowing (p.f.) during each gestation. Of each investigated litter, three piglets were included with the indicated birth order. Colostrum samples were collected from three different teats from each dam. By determining birth weight and 24-h body weight, colostrum uptake (Cl24) was quantified as described by Theil et al. [18]. Blood and milk samples were collected at the indicated time points to read out levels of IgG and IgA binding to *S. suis cps*2 (strain 552) and survival of this strain in blood in vitro (bactericidal assay, BA). In two litters (N and U) last born piglets are missing in the analysis because one piglet was crushed by the sow in the first week of life (old sow) and one died peracutely in the weaning area (middle-aged sow). In a further piglet data on Cl_24_ is missing. The Figure was generated using Biorender

This study was conducted prospectively to explore parameters influencing bactericidal immunity (Fig. 1). The case number was based on the availability of animals and resources in the lab. The influence of various factors associated with colostrum uptake on bactericidal immunity against *S. suis* has not been investigated in previous studies. Accordingly, it was not possible to make plausible assumptions necessary for a formal determination of case numbers. Suckling piglets born by different sows or gilts were not commingled on this farm during the trial. Figure 1 depicts the time points of vaccination and sampling of piglets.

Vaccination was conducted with an autogenous multivalent bacterin applied in a volume of 2 mL i.m.. The autogenous vaccine included among other bacteria (see above) formaldehyde-inactivated *S. suis* 550 and 552 (see below) as well as 10% Emulsigen^®^ (oil-in-water emulsion, MVP Adjuvants, Phibro Animal Health Corporation, Teaneck, NJ, USA) as an adjuvant. All gilts were prime-boost vaccinated with this autogenous vaccine during quarantine and also boost-boost vaccinated 6 and 3 weeks before farrowing (Fig. 1A). All sows received a single boost vaccination 3 weeks before each farrowing. Sows between their second and fourth gestation were defined as middle-aged sows. Old sows were in the fifth to eighth gestation. The gestation of each sow included in this study is specified in Supplementary Material 1. The samples were collected in four different groups of dams. In each group, two gilts, two middle-aged sows and two older sows were sampled. Accordingly, the piglets investigated in this study were born and raised in four, partially overlapping periods. All samples investigated in this study were collected between the 23rd of October 2023 and the 10th of July 2024. Still, the different groups of piglets were represented with the same number of piglets in each run. Very weak neonatal piglets with a body weight below 800 g were excluded from this study.

### Measurement of body weight and tagging of the piglets

Every neonatal piglet born by dams participating in this trial was dried with a towel directly after birth and numbered with a marker according to the birth position. Afterwards they were weighed in a cotton bag with a suitcase scale. After putting the piglets back to the dam, the cotton bag was weighed again empty to subtract its weight from the total weight of the piglet and the cotton bag (determination of birth weight). Farrowing was completed in all cases within 300 min (mean 229 min). At the end of the farrowing, the dam’s first-born, middle-born and last-born piglets were identified and marked with numbered ear tags. This weighing procedure was repeated for every measurement of the piglet’s bodyweight in the farrowing area. After weaning, the piglet’s weight was determined with a personal scale and the weight of the investigator holding the piglet was determined (the weight of the investigator was subtracted afterwards).

### Collection of colostrum and determination of colostrum intake

Within 15 min after birth of the first piglet, colostrum samples were collected from three teats (the first on the left side, the fourth on the right side, and the last on the left side) of each gilt and sow included in this study. Teats were cleaned and milked to obtain a volume of approximately 5 mL of colostrum per teat. Colostrum was cooled immediately and frozen at -80 °C in aliquots within 24 h after collection. The samples of the three teats of each gilt or sow were pooled with equal shares for the ELISA measurements.

Immediately after birth and precisely 24 h later, the piglets of this study were weighed to determine the birth and the 24-h body weight, respectively. The colostrum intake in the first 24 h (Cl_24_) was calculated using the equation published by Theil et al. [18], which was also used in the previous *S. suis* study, which included quantitative data on colostrum intake [19].

### Collection of milk samples

Milk samples were collected within 24 h after weaning by milking three teats of each gilt or sow. The sows were already separated in the breeding centre into their individual stalls, and the samples were taken as a pool from all three teats. The samples were cooled immediately and frozen at -80 °C within two hours of collection.

### Bacterial strains and growth conditions

*S. suis* strain 550 and 552 were isolated from the joints of a diseased suckling piglet and weaning piglet in this herd in 2019 and 2016, respectively.

Streptococci were cultured in Todd Hewitt Broth (THB) and plated on Columbia agar plates with 5% sheep blood. Strains were stored at -80 °C with 20% glycerine added.

### Genotyping and MLST analysis of *S. suis*

Profiling of virulence-associated genes such as *sly*, *mrp* and *epf* by MP-PCR and sequencing of the *cps*K gene to differentiate between *cps*2 and *cps*1/2 as well as *cps*1 and *cps*14 was conducted as described [20, 21]. The sequence type (ST) was determined by MLST [5].

### Bactericidal assay

Blood was collected in heparinized tubes at an age of 2, 6 and 10 weeks of life in this study to conduct bactericidal assays. For this, 1 × 10^6^ colony forming units (CFU) of *S. suis* strain 550 or 552 was added to 500 µL heparinized (16 I. U. heparin/ml) blood. Through a serial dilution of the sample and plating on blood agar plates, the initial specific bacterial load was determined (t = 0 min). In vitro infected blood samples were incubated on a rotator for 120 min at 37^°^C. Serial dilutions and plating of the samples were repeated to determine CFU at t = 120 min. Survival factors were calculated through division of CFU at t = 120 min by CFU at t = 0 min.

### Detection of IgG and IgA binding to *S. suis* cps2 strain 552 in serum, colostrum and milk samples

Levels of IgG and IgA binding to immobilized and inactivated *S. suis* strain 552 were determined in ELISA as described below. Hereafter, these levels are also referred to as anti-*S. suis cps*2 IgG and IgA levels or specific IgG and IgA levels, respectively. Each sample was measured in a duplicate series of four dilutions. All ELISA measurements were standardized by including a duplicate series of 7 dilutions of the same standard serum on each plate. A serum of a pig that was prime-boost vaccinated with a *cps*2 bacterin in a previous study served as standard serum defining 100 ELISA units in the IgG and IgA ELISA. This serum (#4515) is known to mediate opsonophagocytosis of different *S. suis cps* [22, 23]. Optical densities were converted to antibody levels through log linear regression analysis. The ELISA units for each sample were defined as the mean of the calculated units for each of the four dilutions of the two series. These calculations took into account the different dilution factors in the wells of the ELISA plate.

Nunc MaxiSorp™ flat-bottom plates (Fisher Scientific, Nunc A/S, Roskilde, Denmark) were coated with 0.2% formaldehyde-inactivated bacteria of strain 552 overnight at 4^°^C. Plates were blocked with 0.5% bovine serum albumin (BSA) and 0.1% gelatin in PBS. After washing, serum samples were added in duplicates serially diluted in PBS with 0.5% bovine serum albumin (BSA), 0.1% gelatin and 0.05% Tween 20. For this, serum samples of piglets were diluted in series ranging from 1:12.5 to 1:50. In contrast, colostrum was diluted with PBS in series ranging from 1:800 to 1:3200. Preliminary measurements revealed that these dilutions of sera and colostrum samples result in values within the linear range of the standard serum. In numerous cases it was necessary to repeat measurements with a different dilution series to obtain values within the linear range of the ELISA. Prior to dilution, colostrum was carefully rotated at room temperature to obtain a homogenized solution that allows accurate pipetting.

For detection of porcine IgG binding to *S. suis cps*2, a secondary polyclonal peroxidase-conjugated goat anti-pig IgG (A100-105 P, Bethyl, Montgomery, Texas, 1:1000) was used. A pool of 71 sera collected from the piglets in week four was used as a positive control. Plates were developed using 2.2-azino-di-(3-ethylbenzithiazoline sulfonate) (ABTS, Roche, Merck, Darmstadt, Germany) and H_2_O_2_ as the substrate. Absorbance was measured at 405 nm. Washing steps and incubation periods were conducted as described previously [24].

For detection of IgA binding to *S. suis cps*2, a peroxidase-conjugated goat anti-pig IgA (A100-102 P, Bethyl, Montgomery, Texas, 1:5000) was used. A serum of a convalescent pig experimentally infected with *S. suis* within a previous study was used as a positive control. Plates were developed using 3,3′,5,5′-3,3,5,5-tetramethylbenzidin (Merck, Darmstadt, Germany) and H_2_O_2_ as the substrate. The reaction was stopped with 1 M sulphuric acid. Absorbance was measured at 450 nm [22, 23]. 

### Detection of total IgG

A competitive ELISA was conducted for measurement of total IgG. Therefore, Nunc MaxiSorp™ flat-bottom plates (Fisher Scientific, Nunc A/S, Roskilde, Denmark) were coated with 0.1 µg/ well of purified porcine IgG for one hour at room temperature. After washing, serum or colostrum samples were added in duplicates and serially diluted in PBS with 0.1% Tween 20 starting from 1:1000 or 1:4000, respectively. Additionally, 1 ng of peroxidase-conjugated protein G (101223, Invitrogen™, Camarillo, California) was added to each well. Following an incubation of one hour at room temperature, plates were washed, developed and measured as described above for IgA detection. Purified IgG from a pool of porcine sera served as standard. Purification of IgG was conducted using affinity chromatography with protein G (17040501, Cytiva Germany, Freiburg, Germany) combined with size-exclusion chromatography through HiLoad^®^ 16/600 Superdex^®^ 200 Prep Grade columns (GE28-9893-35, Sigma-Aldrich Chemie GmbH, Taufkirchen, Germany).

### Detection of IgA binding to surface-associated antigens of *S. suis cps*2 strain 552 in serum of piglets

A further IgA ELISA with serum samples of middle born piglets of different litters drawn at 2-, 6- and 10-weeks of age was conducted using a different antigen preparation and a monoclonal anti IgA antibody. Wells were coated with 4 µg/mL surface-associated protein released by ultrasonic treatment of *S. suis cps*2 strain 552 bacteria inactivated with 0.4% peracetic acid. A monoclonal peroxidase-conjugated antibody directed against porcine IgA (clone K61 1 B4, MCA6386A, Bio-Rad Laboratories, Munich, Germany) was used in a concentration of 200 ng/mL as detection antibody. Otherwise, the ELISA was conducted and developed as described above for the IgG or IgA ELISA using polyclonal detection antibodies.

### Statistical analysis

Levels of antibodies and bacterial survival factors were transformed to obtain approximately normally distributed data. In the case of specific antibody levels measured in ELISA, data was logarithmized with log(1 + x). In case of survival factors of *S. suis* strain 552 determined in the bactericidal assay, Box-Cox-transformation with λ = 0.2 was conducted (Supplementary Material 2).

Data are presented as mean ± standard deviation (SD). Inter-group comparisons were performed using ordinary one-way or repeated measures ANOVA with Tukey’s multiple comparisons range test for post-hoc analyses. We used a one-sample t test on the Pearson coefficients for ordinary linear regression analyses (GraphPad Prism 10, USA). P values < 0.05 were considered statistically significant.

Linear mixed-effects models (LMMs) were applied to systematically explore the effect of different parameters, e.g. sow parity, CI_24_ or levels of specific immunoglobulins in serum at multiple timepoints on the survival factors of the streptococci. This type of analysis was used to account for the design of the study with the repeated measures and to adequately describe the dependency structure within the data, since three piglets from each dam were included.

LMM analyses were performed in R v4.4.1 using the lme4 package [25]. The transformed survival factor of *S. suis* strain 552 in porcine blood was included as the dependent variable. To assess the effect of time (i.e., piglet age), we first specified a default model M1 as follows: M1: survival factor ~ time + (time | dam/piglet). Here we account for nested random effects of dams and piglets (denoted as dam/piglet) as well as for repeated measures (parameter time). In addition, time was added as a fixed effect, allowing us to extract the main effect of age regardless of individual dams and piglets. Next, to test for the fixed effect of each parameter, two other LMMs were fitted incrementally: M2: survival factor ~ time + parameter + (time | dam/piglet) and M3: survival factor ~ time * parameter + (time | dam/piglet). In M3, the asterisk represents a linear combination of both a fixed effect of the parameter (see M2) and a fixed interaction between piglet age (time) and the parameter (denoted as time: parameter). While in M2 the main effect was considered to be the same at all timepoints, M3 additionally assessed the significance of an interaction between the effect of age and the effect of the parameter.

For each parameter, a pairwise comparison of the models was performed using likelihood ratio tests. A significant difference between M2 and the default model M1 indicates that the parameter had an effect on the survival factor across the whole time period. Likewise, a significant difference between M3 and M2 indicates that an interaction between the parameter and piglet age was supported by the data.

Significance of LMM coefficients was assessed by the lmerTest package [26], which applies Satterthwaite’s method to estimate degrees of freedom and generate p values for LMMs. 95% confidence intervals (CIs) of coefficient estimates were calculated using bootstrapping with 10^5^ replicates.

## Results

### Study design and levels of total IgG and *S. suis* specific IgG in colostrum

**Fig. 2:**
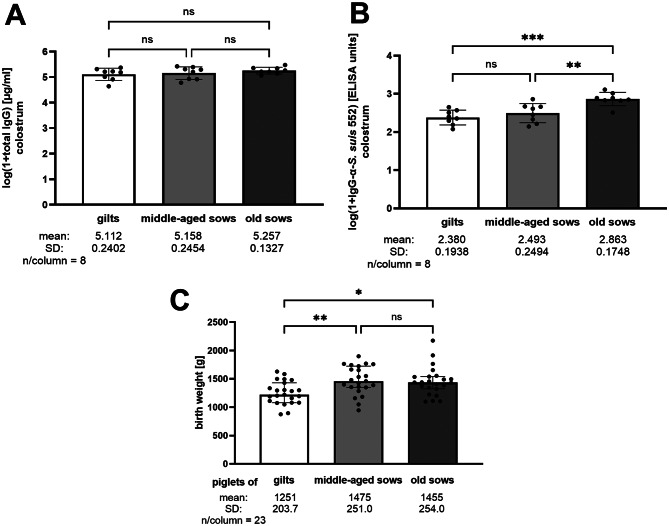
Comparison of total IgG (**A**) and levels of specific IgG binding to *S. suis cps*2 strain 552 (**B**) in colostrum of gilts, middle-aged sows and old sows as well as birth weights of their piglets further investigated in this study (three piglets per dam) (**C**). The vaccination protocol is depicted in Fig. 1. Statistical analyses were conducted with the one-way ANOVA and a subsequent Tukey’s multiple comparisons test. Significant differences are indicated (* *p* < 0.05, ** *p* < 0.01, ***p < 0.001)

This study was conducted in a herd that has not experienced major *S. suis* disease outbreaks in recent years. In this herd, a multivalent *S. suis* vaccine including *cps*1 and *cps*2 strains has been used over 10 years in gilts and sows. We obtained two of these strains, designated 550 and 552, from the company generating the autogenous vaccine and genotyped 550 as *mrp* + *sly* + *epf* + *cps*1 of ST1 and 552 as *mrp* + *sly* + *epf* + *cps*2 of ST1. Bactericidal assays with strain 550 resulted in killing of more than 90% of the streptococci in 88.7%, 100% and 98.6% of the blood samples collected at 2-, 6- and 10-weeks of age (results not shown). As we wanted to explore differences between the piglets in bactericidal immunity, we focused on the *cps*2 strain 552 that showed a much broader range of bacterial survival factors (see below). In this herd, we investigated factors determining levels of maternal antibodies in piglets, the course of *S. suis cps*2 specific IgG and IgA as well as bactericidal immunity in blood of piglets. Gilts and sows were vaccinated with the autogenous multivalent *S. suis* vaccine as outlined in Fig. 1 including a boost vaccination during each gestation (3 weeks pre-farrowing). The study design included comparison of piglets farrowed by gilts, middle-aged sows and old sows. The concentration of total IgG in colostrum hardly differed between the three groups of dams (Fig. 2A). In contrast, colostrum of gilts contained significantly lower anti-*S. suis cps*2 IgG levels than colostrum of old sows (*p* = 0.0004, Fig. 2B), though incoming gilts were prime-boost vaccinated during quarantine and additionally boost-boost vaccinated prior to farrowing. As old sows also had significantly increased specific IgG levels in comparison to middle-aged sows (*p* = 0.0051), our data suggests that boost vaccination of dams with an autogenous *S. suis* vaccine prior each farrowing potentiates increased levels of IgG directed against *S. suis cps*2 with increasing age of sows. Further, the birth weight of piglets of gilts was significantly lower compared to piglets of middle-aged and old sows with a mean of 1251 g ± 203.7 compared to 1475 g ± 251 and 1455 g ± 254, respectively (Fig. 2C).

### Factors associated with the concentration of total IgG and *S. suis* specific IgG levels in suckling piglets

**Fig. 3:**
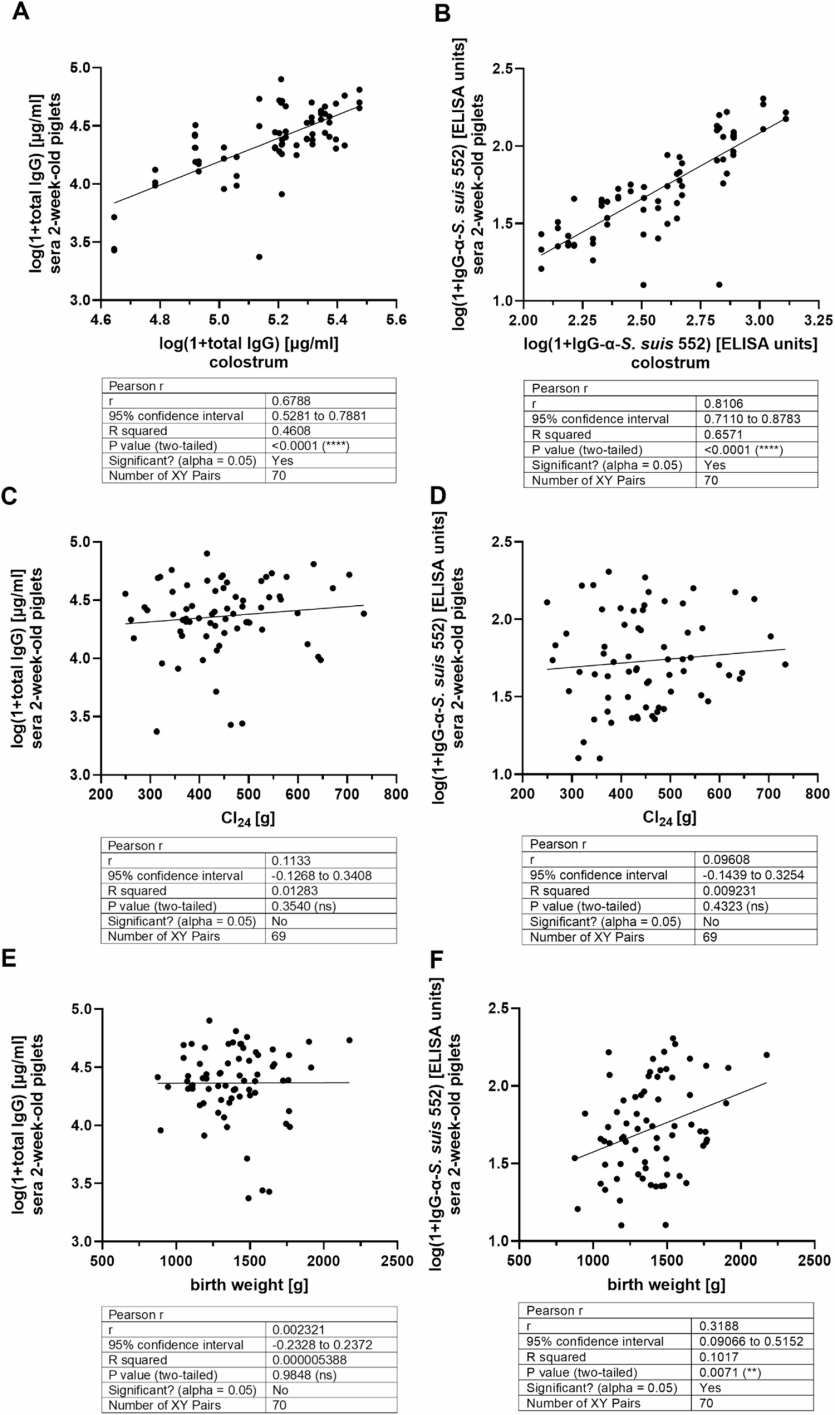
Correlation analyses of the concentration of total IgG and levels of specific IgG in serum of 2-week-old piglets with the concentration of total IgG and specific IgG levels in colostrum (**A**,** B**), with colostrum uptake during the first 24 h (Cl_24_) (**C**,** D**) and with body weight at birth (**E**,** F**). Levels of specific IgG were defined as the levels measured in ELISA using inactivated whole bacteria of *S. suis cps*2 strain 552 as coating antigen. The table below each figure shows the results of the statistical analyses

A previous study [19] showed that colostrum intake and birth weight had a significant positive association with the amount of *S. suis*-specific antibodies in one-day-old piglets. A significant association with antibody levels in colostrum was only found for *S. suis cps*9 but not *cps*2. Here, we also investigated these factors but differentiated isotypes of antibodies and sampled 2-week-old piglets. As shown in Fig. 3A and B, the concentration of total IgG and levels of specific IgG in colostrum correlated with the concentration of total IgG and specific IgG levels in serum of 2-week-old piglets with high correlation coefficients of *r* = 0.68 and *r* = 0.81, respectively. These piglets had taken up between 250 g and 733 g colostrum in the first 24 h of life (Cl_24_) based on our calculation using the equation by Theil et al. [18]. Specifically, first-, middle- and last-born piglets took up a mean of 465 ± 131, 456 ± 100 and 425 ± 85 g Cl_24_, respectively, but differences between these three groups were not significant (first versus middle: *p* = 0.9: first versus last: *p* = 0.4; middle versus last: *p* = 0.6). We did not detect a correlation between Cl_24_ and the concentration of total IgG (Fig. 3C) or specific IgG levels (Fig. 3D) in serum at 2-weeks-of-age in this herd (*p* = 0.35 and *p* = 0.43, respectively). The birth weight of the piglets included in this study ranged from 875 to 2175 g. Specifically, mean birth weights of 1410 ± 250, 1398 ± 276 and 1370 ± 247 g were recorded for first-, middle- and last-born piglets, respectively, without significant differences between these three groups. Birth weight showed a significant correlation with specific serum IgG levels at an age of 2 weeks (*p* = 0.007; Fig. 3F) but not with the concentration of total IgG (Fig. 3E, *p* = 0.98). First born piglets took up 465.4 g (S.D. = 130.8 g) colostrum (Cl_24_) which was higher than Cl_24_ of middle- and last-born piglets with 455.6 g (S.D. = 100 g) and 424.6 g (S.D. 130.8 g), respectively, but differences were not significant (Supplementary Material 3). First-, middle- and last-born piglets showed mean IgG antibody levels against *S. suis cps*2 strain 552 of 1.792 ± 0.2696, 1.746 ± 0.2771 and 1.623 ± 0.3489 on the logarithmic scale, respectively, but these differences were also not significant (Supplementary Material 4). In summary, our data indicates that the concentration of total IgG and the level of specific IgG in suckling piglets is to a large extent determined by the concentration of total IgG and specific IgG levels in colostrum in this herd, respectively. Furthermore, this study confirms that birth weight is positively associated with specific IgG levels at 2-weeks of age and that the age of the dams has a strong impact on both these factors in this herd.

### Levels of IgA binding to *S. suis cps*2 in colostrum, milk and serum

**Fig. 4:**
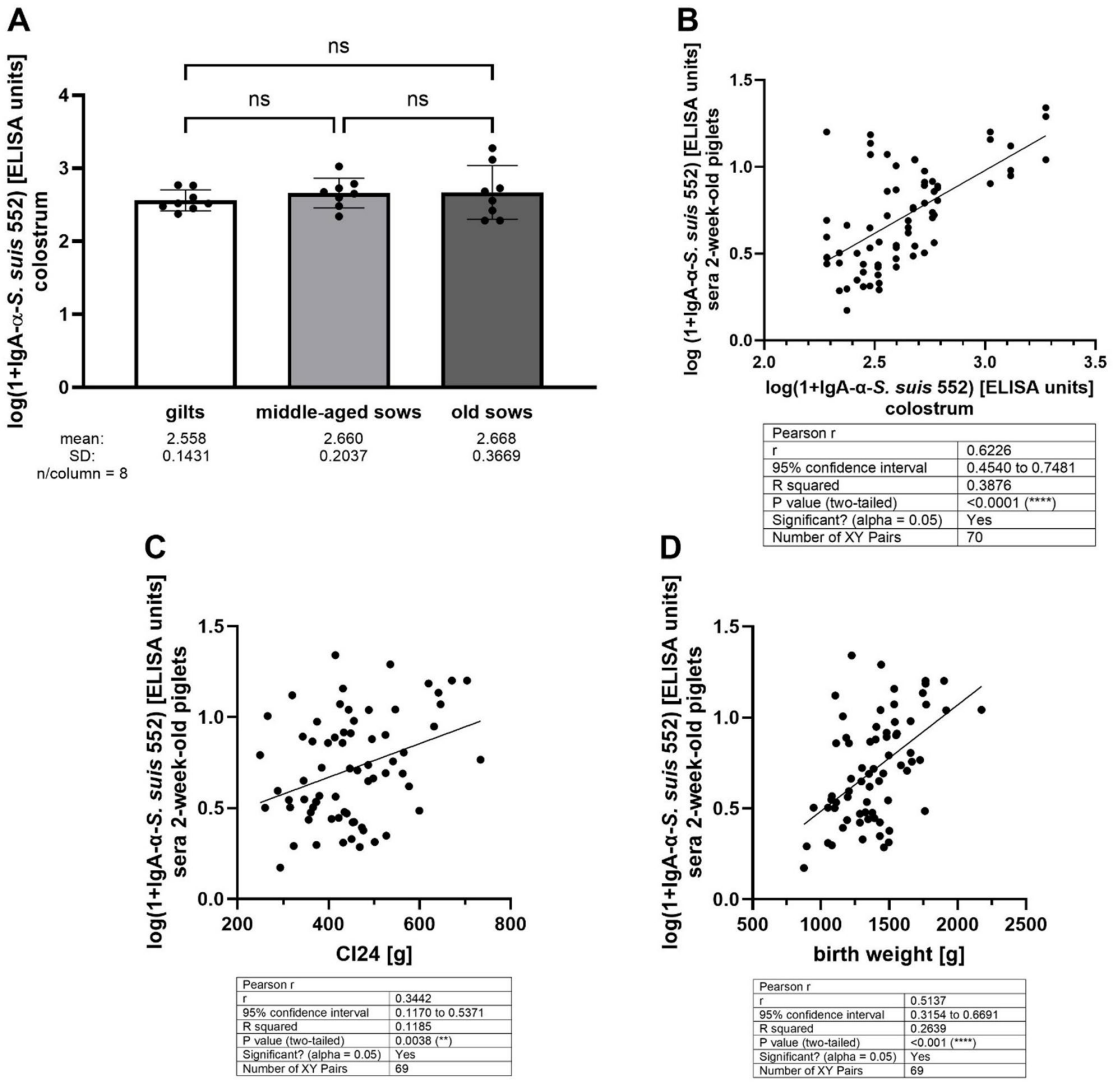
Levels of IgA binding to *S. suis* c*ps*2 strain 552 in colostrum of dams vaccinated with an autogenous vaccine as shown in Fig. 1. **A** Comparison of specific IgA levels in colostrum of gilts, middle-aged sows and old sows. **B** Correlation analysis of specific IgA levels in colostrum and respective levels in serum of 2-week-old piglets of these dams. **C** and **D** Correlation analyses of *S. suis cps*2 specific IgA levels in sera of 2-week-old piglets and colostrum uptake in the first 24 h (**C**) and body weight at birth (**D**)

Porcine colostrum includes also substantial amounts of IgA [27]. In this herd, colostrum of investigated gilts, middle-aged sows and older sows showed similar mean levels of specific IgA between 2.5 and 2.6 log (1 + IgA-α-*S. suis* 552) ELISA units without significant differences between these three groups (Fig. 4A). These three groups had also comparable levels of specific IgA in milk collected after weaning with mean values between 2.1 and 2.3 log (1 + IgA-α-*S. suis* 552) ELISA units (Supplementary Material 5). The levels of colostral IgA binding to *S. suis* 552 showed a high correlation with respective serum IgA levels in 2-week-old piglets (*r* = 0.62; Fig. 4B). Furthermore, specific IgA levels in these suckling piglets correlated also significantly with the amount of colostrum taken up (Cl_24_) and body weight at birth (Fig. 4C and D, respectively). First-, middle- and last-born piglets showed mean serum IgA antibody levels against *S. suis cps*2 (strain 552) of 0.7738 ± 0.2749, 0.6917 ± 0.2824 and 0.6643 ± 0.3131 on the logarithmic scale, respectively. Differences between the three groups were not significant (first versus middle: *p* = 0.6; first versus last: *p* = 0.4; middle versus last: *p* = 0.9). In summary, specific IgA levels in colostrum and milk were very similar between the three age groups of dams and serum IgA levels in suckling piglets depended on both, the quantity of colostrum uptake and the respective level in colostrum.

### Longitudinal study of specific IgG, IgA and bactericidal immunity in blood of 2-to 10-week-old piglets

**Fig. 5:**
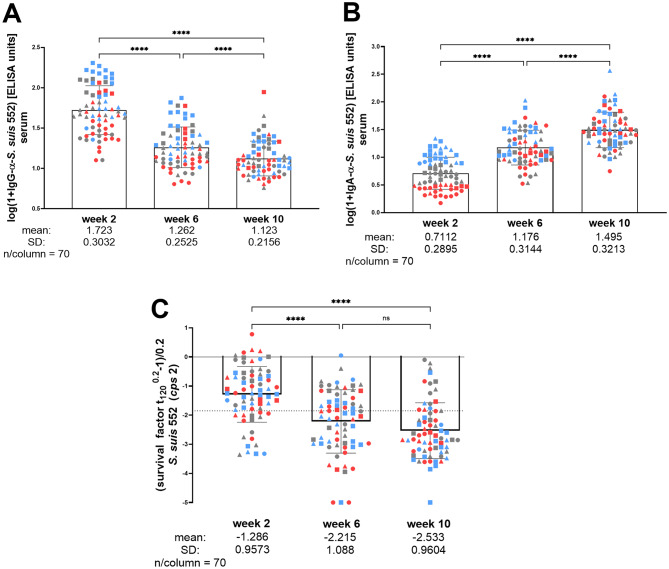
Levels of specific serum IgG (**A**) and IgA (**B**) antibodies binding to *S. suis cps*2 strain 552 in 2-, 6- and 10-week-old piglets as well as survival (**C**) of this strain in heparinized blood drawn from the same animals at the same time points. (**A**,** B**) Specific antibody levels were measured in ELISA using inactivated bacteria of this strain as an immobilized antigen. Values were transformed as indicated to obtain approximately normally distributed data (Supplementary Material 2). Values of piglets were marked with different forms according to the ranking of their specific IgG levels against *S. suis cps*2 in the 2nd week of life: Squares, triangles and circles refer to the upper, middle and lower third of respective values of specific IgG levels. A color code was used to rank the particular IgA levels in the 2nd week of life: Blue, grey, and red refer to the upper, middle, and lower third of respective values of specific IgA levels. **C** The survival factor was transformed as indicated. A value of zero indicates an unchanged bacterial load and corresponds to an original survival factor of 1. The dotted line indicates a transformed value of -1.85, corresponding to an original survival factor of 0.1. Accordingly, all piglets showing values below this dotted line killed more than 90% of the streptococci added to the heparinized blood sample

We investigated how specific IgG and IgA levels developed in these piglets in the following 8 weeks of life. Furthermore, we read out bactericidal immunity in porcine blood in vitro at the same time points to test the hypothesis that the expected drop of maternal antibodies in the following weeks of life is associated with a decrease in the killing efficiency of streptococci in blood. Specific IgG declined significantly from a mean of 1.72 ± 0.30 to 1.26 ± 0.25 logarithmized ELISA units in serum of piglets between the 2nd and 6th week of life (Fig. 5A). We even observed a further significant, though less prominent, decline to a mean of 1.12 ± 0.22 log (1 + α-IgG *S. suis* 552) ELISA units from the 6th to the 10th week of life (Fig. 5A). As specific IgG levels showed variation of more than one log in the 2nd week of life, we divided the animals into three categories according to these values with circles referring to the lowest third of maternal antibody levels (Fig. 5A). Though single piglets showed an increase of specific IgG levels between the 6th and the 10th week of life, specific IgG levels declined in the majority of piglets until the end of the observation period of 10 weeks. This also applies to the lowest third of maternal IgG levels.

In contrast to IgG, we observed a prominent and highly significant increase in serum IgA binding to *S. suis cps*2 between the 2nd and 6th week of life and between the 6th and 10th week of life (Fig. 5B). At 2 weeks but not 6 and 10 weeks of age, specific serum IgA levels correlated with specific IgG levels (Supplementary Material 6). To verify the increase of IgA recognizing surface-associated antigens of *S. suis cps*2, a further ELISA was conducted with sera of middle-born piglets of 9 litters. In contrast to the ELISA that was used to read out the data shown in Fig. 5B, surface-associated antigens released by ultrasonic treatment were used as coating antigen and a monoclonal anti-IgA detection antibody was used. As shown in Supplementary Material 7, this independent ELISA confirmed the significant increase of IgA recognizing *S. suis* antigen during the observation of the piglets between the 2nd- and 10th week of life.

In the bactericidal assays, freshly collected blood of the piglets was infected with the *cps*2 *S. suis* strain 552 in vitro to assess bacterial survival in the presence of numerous active parts of the host immune system, such as immunoglobulins, complement, and neutrophilic granulocytes. In the case of virulent *S. suis* strains such as 552, killing in this assay is mainly elicited by antibody-mediated opsonophagocytosis [23]. We conducted the bactericidal assay with blood drawn from 2-, 6- and 10-week-old piglets using the same infection dose of *S. suis cps*2 strain 552. Reduction of the number of streptococci to less than 20% of the starting inoculum within the 2 h incubation period was recorded for 40.9%, 75.7% and 82.9% of the blood samples drawn from 2-, 6- and 10-week-old piglets, respectively. Due to right skewed distributions, survival factors at each time point were Box-Cox-transformed with λ = 0.2 before statistical analysis using parametric methods (Supplementary Material 2). This resulted in a value of 0 for an unchanged bacterial load (100% survival) and values of -1.38 and − 1.85 for killing 80% and 90% of the streptococci, respectively. As shown in Fig. 5C, the streptococcal survival factor significantly decreased between the 2nd, 6th and 10th week of life with mean values of -1.29 ± 0.96, − 2.22 ± 1.09 and − 2.53 ± 0.96, respectively. Accordingly, most piglets showed only moderate killing of the streptococci at 2 weeks of age with a remaining bacterial load in the blood above 10% of the starting inoculum. In contrast, more than 80% and 90% of the streptococci were killed in the majority of blood samples collected from the same piglets at an age of 6 and 10 weeks, respectively (Fig. 5C). Thus, the decrease of maternal IgG antibodies between the 2nd and 6th week of life is overall not associated with less efficient killing of *S. suis cps*2 in blood of these piglets.

Linear mixed-effects models were applied to explore the effect of different variables on killing of *S. suis cps*2 in blood of piglets over the observation period ranging from the 2nd to the 10th week of life. Colostrum uptake, birth weight, parity of the dam, birth position, specific IgG and IgA levels in colostrum and specific IgG levels in serum of piglets were all not associated with the survival factor of the streptococci (Supplementary Material 8). Only specific IgA levels in serum of the piglets and body weight as measured at the 2nd, 6th and 10th week of life showed a statistically significant effect on bacterial survival in blood of these piglets (Table 1). While body weight showed a positive, time independent effect on bacterial survival, increased specific IgA levels in serum showed a negative effect on survival of the streptococci in porcine blood. In addition, the latter effect was time-dependent as the data supported an interaction between specific IgA levels in serum and piglet age. We also compared the survival of *S. suis cps*2 552 in blood of gilts, middle-aged sows and old sows as well as of first-, middle- and last-born piglets at different time points. Differences between these three groups were not significant (Supplementary Material 9) with one exception: At 6 weeks of age, last born piglets showed a significantly lower bacterial survival factor than first-born piglets.

**Fig. 6:**
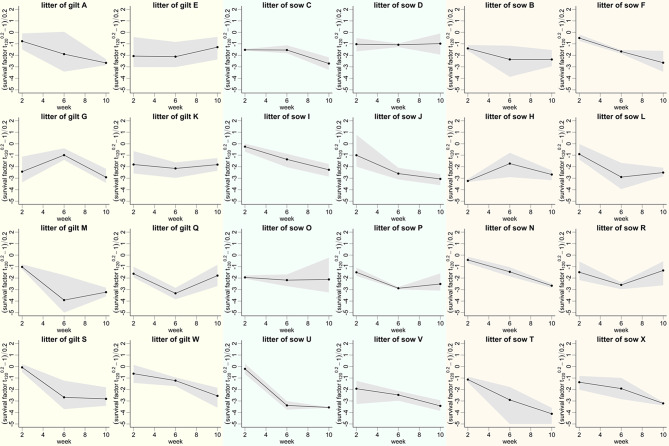
Time course of Box-Cox transformed survival factors of *S. suis cps*2 strain 552 in blood samples of piglets of different litters. Blood was collected for this assay from the same three littermates at 2, 6, and 10 weeks of age. In sows N and U, only two littermates were investigated in bactericidal assays because one selected littermate died early. Filled circles depict the mean survival factor, while the gray shadows indicate the range of the data at each timepoint. The two columns on the left, in the middle, and on the right show the data of the litters of the gilts, middle-aged sows, and old sows, respectively

Figure 6 shows the Box-Cox-transformed survival factors for the different litters (*n* = 24) separately. Only in the litters of gilt G and sow H an increase in the survival factors of streptococci between the 2nd and 6th weeks of life was recorded. Of note, the littermates of these two dams showed very efficient killing of the streptococci in blood collected at 2-weeks-of-age (more than 90% killing) compared to the other piglets. Overall, substantial differences in the course and divergence of streptococcal survival factors in blood between litters were recorded. The data for several litters (C, D, F, I, O, P, and Q) showed minimal deviation among the three investigated littermates until the 6th week of life. However, substantial variations were observed in other litters (A, B, E, H, M, T, V). In numerous litters the variation in survival factors was more pronounced at 6 weeks than at 2 weeks of age (A, B, E, H, M, S, V).

In summary, killing of *cps2 S. suis* strain 552 at 2, 6, and 10 weeks of age did not correlate with any of the investigated parameters related to maternal immunity, such as colostrum uptake and specific IgG and IgA levels in colostrum. In 2- to 10-weeks old piglets, serum IgA binding to *S. suis* shows a prominent increase and a statistically significant effect on killing of the streptococci in blood.

**Table 1:** Coefficients of linear mixed-effects models for parameters weight_t2,t6,t10_ and serum α-*S. suis* IgA linear on survival of *S. suis cps*2 strain 552 in blood of piglets drawn at 2, 6 and 10 weeks of age^a^

Parameter	Model^b^		Estimate β	95% CI	SE	*P* value
		intercept	-0.9623	[-1.3304, -0.5929]	0.1884	< 0.001
weight_t2,t6,t10_^c^	M2	time	-0.2647	[-0.3716, -0.1583]	0.0542	< 0.001
		weight_t2,t6,t10_	0.0425	[0.0062, 0.0786]	0.0184	0.0219
serum α-*S. suis* IgA	M3	intercept	-0.1626	[-0.9240, 0.5918]	0.3821	0.6713
		time	-0.2436	[-0.3875, -0.0993]	0.0724	< 0.001
		IgA-α-*S. suis* 552 serum	-0.5023	[-0.8513, -0.1531]	0.1756	0.0048
		time: IgA-α-*S. suis* 552	0.0510	[0.0029, 0.0990]	0.0242	0.0361

a Coefficients of linear mixed-effects models for parameters birth position, parity, birth weight, weight t24, CI_24_, colostrum α-*S. suis* IgG, serum α-*S. suis* IgG- and colostrum IgA-α-*S. suis* are given in the Supplementary Material 5. A significant effect on survival of *S. suis* in porcine blood was not recorded for these parameters

b For weight_t2,t6,t10_ a statistically significant effect on the survival factor across the whole time period could be demonstrated (model M2). Serum α-*S. suis* IgA- was also found to have a significant effect on the survival factor. In addition, an interaction between piglet age and the parameter was observed, indicating a time-dependent effect (M3)

c Weight gain between weeks 2 and 10 is designated as weight_t2,t6,t10_

## Discussion

Different research groups have shown that vaccination of gilts or sows pre-farrowing with autogenous *S. suis* bacterins elicits increased IgG levels against the homologous strain [10–12]. Suckling piglets of vaccinated gilts or sows have elevated levels of IgG binding to these vaccine strains compared to unvaccinated piglets within the same herd. Whether IgA levels against *S. suis* in piglets are also influenced by bacterin vaccination of dams is not well investigated. In a recent study, we did not record an effect of bacterin vaccination pre-farrowing on serum IgA levels of piglets against *S.suis cps*1 [28]. As the analysis presented here did not include a group of non-vaccinated piglets, it is unclear if IgG and IgA levels were increased due to vaccination. Nevertheless, the former appears very likely due to the very high levels of specific IgG in 2-week-old piglets, the results of other studies with autogenous vaccines using the same adjuvant and similar vaccination schedules [10] and the fact that specific IgG levels were higher in colostrum of old sows which have gone through numerous boost vaccinations.

One remarkable finding of this study is the decline of specific IgG until the 10th week of life. The results shown in Fig. 5A suggest that most piglets in this herd do not undergo an active adaptive immune response with prominent secretion of IgG binding to *S. suis* during the critical nursery period. This observation agrees with a study showing a decline of IgG binding to MRP until the 8th week of life in piglets of sows also vaccinated with a *S. suis cps*2 bacterin pre-farrowing [10]. MRP is one of the main immunogens on the surface of virulent *S. suis cps*2 strains of CC1 [29, 30]. Likely, some of the antibodies detected in the current study were also directed against MRP. One possible explanation for the ongoing decline of IgG levels after weaning is that maternal immunity inhibits the adaptive immune response with the generation of plasma cells secreting IgG antibodies that are binding to *S. suis*. Of note, we only measured levels of IgG binding to the surface of immobilized, whole bacteria and not to single antigens. Accordingly, the results of this study do not exclude an increase of IgG binding to other *S. suis* antigens in the observation period. This might include IgG antibodies against suilysin which is secreted and very immunogenic [30, 31]. Different experimental studies with weaning piglets have demonstrated an increase of specific IgG levels between 4 and 8 weeks of life after prime-boost vaccination of weaning piglets with *S. suis* bacterins [32–34] or experimental infection [30, 35]. Therefore, it can be excluded that piglets of this age are generally incapable of exerting an IgG response against surface antigens of *S. suis*. Another explanation is that piglets were not infected with *S. suis cps*2 in a way that resulted in a systemic IgG response. We chose a herd with remarkably few problems with *S. suis* diseases so that the infection pressure might have been relatively low. Nevertheless, it is striking that in the same piglets a highly significant increase of serum IgA binding to *S. suis cps*2 was observed (Fig. 5B). This might demonstrate the capability of inducing an active adaptive immune response against this pathogen between the 2nd and 10th week of life [21, 35, 36].

Vreman et al. [19] recently described a decrease of maternal antibodies binding to *S. suis* until the 18th day of life and an increase of specific antibodies afterwards. Based on the results of this and other studies [10], it appears pretty likely that the described course of antibody levels recorded by using a secondary antibody against the light chain of immunoglobulins is a result of an overlap of declining maternal IgG and increasing IgM and IgA levels during the nursery phase. The results of this study indicate that the decline of maternal IgG binding to *S. suis* might extend until weeks after weaning in piglets fostered by vaccinated dams.

In agreement with our results, *S. suis cps*2 specific antibody levels in suckling piglets correlated with birth weight in the previous study [19]. However, in comparison to specific antibody levels in colostrum, Cl_24_ showed a substantially higher Pearson correlation coefficient with *S. suis cps*2 specific antibody levels in suckling piglets in this former study, which contradicts our findings for specific IgG (Fig. 3D). The uptake of colostrum in the first 24 h, Cl_24_, was determined the same way in both studies. Furthermore, colostrum was sampled immediately after birth of the first piglet in both studies. Therefore, it appears unlikely that determination of Cl_24_ or collection of colostrum explains the difference between the two studies. We speculate that there might be important differences between farms regarding these results. In the case of a good supply of colostrum, as in the presented case with values above 250 g in almost all piglets [36, 37], differences in Cl_24_ might have a lower impact on levels of serum IgG antibodies binding to inactivated streptococci in suckling piglets (Fig. 3D).

Birth weight of piglets and specific colostral IgG levels but not the concentration of total IgG in colostrum were significantly increased in multiparous sows in comparison to gilts (Fig. 2). This presumably explains why birth weight correlated significantly with levels of specific IgG in sera of 2-week-old piglets but not with the concentration of total IgG in these sera, although both parameters showed correlation with respective values measured in colostrum (Fig. 3).

It was unexpected that we did not record increased IgG and IgA levels (results not shown) or bactericidal immunity in first- or middle-born piglets compared to last-born piglets. Of note, it is known that antibody levels in colostrum drop already 6 h after birth of the first piglet [38]. As Cl_24_ was above 250 g in all piglets and last-born piglets were born within 300 min (mean 229 min) after the first piglet, colostrum supply was not a limiting factor in this investigated cohort of piglets.

The term immunity gap describes the period, when the piglet`s immunity is not developed enough to compensate for the decline of maternal antibody levels, and susceptibility to infectious diseases is substantially increased. This model has also been proposed for *S. suis* [19]. However, it appears difficult to determine this period by measuring antibody levels, as shown here: Killing of the streptococci in blood became more efficient between the 2nd and 6th week of life, though specific IgG levels declined. As we found a significant correlation between specific IgG levels in piglets and colostrum, this decline of specific IgG reflects the decline of maternal IgG. Corsaut et al. [39] recorded a decrease of opsonizing antibodies mediating killing of *S. suis cps*7 in serum between the 7th and 18th day post-partum in piglets of sows vaccinated with an autogenous bacterin pre-farrowing. As we conducted the bactericidal assay for the first time at 2 weeks, we cannot exclude that killing of *S. suis cps*2 was also more efficient earlier, such as the 7th day, due to higher levels of maternal antibodies.

Furthermore, levels of opsonizing antibodies might have declined after the 2nd week for some time to finally increase again, leading to the very efficient killing in the 6th and 10th weeks. Despite these uncertainties, the linear mixed-effects model analysis results show that levels of IgG binding to immobilized *S. suis cps*2 do not affect killing of the streptococci in the bactericidal assay. This agrees with the observation that piglets with high levels of IgG antibodies against surface-associated antigens of *S. suis* are often highly susceptible to *S. suis* disease as shown in different challenge experiments [22, 32].

The ELISA used in this study to detect IgG against *S. suis* most likely detects different subtypes of IgG as a polyclonal anti-pig IgG antiserum is used. The 8 recently described subclasses of porcine IgG presumably differ in their Fc-mediated functions such as C1q binding and complement activation important for opsonophagocytosis of *S. suis* [40, 41]. Accordingly, one might speculate that a correlation between levels of a specific IgG subtype against *S. suis* and killing of streptococci in porcine blood might exist. For detecting pig IgG in ELISA, one might also use two commercially available murine antibodies that were raised against different preparations of porcine IgG designated “IgG1” and “IgG2”. However, the specificity regarding the current IgG subtype definition by Zhang et al. [41] is unclear. Nevertheless, the ratio of “IgG1” to “IgG2” was found to be shifted towards “IgG1” in piglets vaccinated with a *S. suis* bacterin including Emulsigen^®^-D as adjuvant. In contrast, a bacterin with Montanide™ ISA 61 VG induced a more balanced ratio [33]. As only the latter was associated with protection in this previous study, the authors postulate that Emulsigen^®^-D, the adjuvant used here, is unsuitable for *S. suis* bacterins. Both, Montanide™ ISA 61 VG and Emulsigen^®^ are oil emulsions. Whereas oil-in-water adjuvants like Emulsigen^®^ can lead to strong short-term antibody responses, water-in-oil adjuvants like Montanide™ ISA 61 VG elicit long term immunity due to a greater depot effect [42]. The immune response of the latter is humoral as well as cellular. Further, water-in-oil adjuvants are associated with a stronger IgG2 response and thus higher bacterial opsonophagocytosis in mice [33, 43]. However, it remains to be confirmed that the ratio of specific “IgG1” to “IgG2” in pigs also correlates with the level of opsonizing activity.

The combination of data from different litters and littermates challenges statistical analysis of the data presented here. On the one hand, littermates take up the same colostrum with a distinct level of antibodies and other ingredients presumably justifying the treatment as paired samples or even biological replicates. The strong correlation of specific IgG and IgA levels in 2-week-old piglets with the level in colostrum supports this argument. On the other hand, factors like colostrum uptake, birth position, body weight and development of immunity were considered likely to influence the read-out parameters investigated in this study. This study confirms that factors like colostrum uptake are relevant for the readout parameters such as IgA levels at 2 weeks of age. We decided to treat each piglet as an individual in our analysis because we observed a high level of data divergence in some litters that covered almost the complete range of data of the entire cohort. However, we chose a design of sample collection that ensures that each litter is represented by only three samples at each time point and that there is no bias towards single litters.

In our mixed-effects model (Table 1) we identified only one factor in addition to time that is significantly associated with reduced survival of *S. suis cps*2 in porcine blood: the level of serum IgA binding to *S. suis cps*2. This was surprising as serum IgA has not yet been described as an important readout parameter in *S. suis* studies. Care should be taken about the claim that serum IgA plays a crucial role in opsonophagocytosis of *S. suis cps*2 in porcine blood. Further studies are necessary to investigate the role of specific IgA in the bactericidal assay. As IgA levels significantly increased during the observation period, specific serum IgA was the only parameter investigated that reflected the development of the piglets’ immune system during the observation period. It is known that IgM opsonizing *S. suis* is increasing after weaning [24, 44, 45]. Accordingly, IgM is likely to play an important role in the killing of streptococci observed in blood samples drawn at 6 and 10 weeks of age in this study. One might argue that *S. suis* specific serum IgA levels are linked to levels of IgM binding to *S. suis* and that this putative linkage determined results of the mixed-effect model. It was not in the scope of this study to investigate such a linkage, but there is currently no clear rational why IgA levels should be directly linked to IgM levels: One plasma cell secretes only one specific immunoglobulin (IgM, IgA or IgG). Non-conventional, CD21^−^ B1-like cells are an important source of IgM binding to *S. suis* in 8-week-old piglets [45]. This B cell subpopulation increases after weaning and shows a strong induction of IgM secretion upon toll-like receptor activation [45, 46]. However, the characterization of B-cells secreting serum IgA antibodies binding to *S. suis* after weaning awaits further investigations. In any case, the data presented in Fig. 6 indicates that in this herd, development of the piglets’ immune system is crucial to obtain the high level of bactericidal immunity against *S. suis cps*2 at 6 weeks of age.

A previous study demonstrated that piglets born later after a longer farrowing duration take up significantly less colostrum than early born piglets [47]. Furthermore, it is known that the IgG concentration in colostrum declines within hours after farrowing [38]. Accordingly, last born piglets receive presumably lower amounts of maternal IgG. Though Cl_24_ and the level of specific IgG in serum of 2-week-old piglets born last was lower in comparison to respective values of middle- and first-born piglets, differences were not significant in this study. High levels of maternal IgG antibodies might be associated with a longer period of inhibition of adaptive immune responses in piglets. Accordingly, piglets with lower maternal IgG levels might go through an earlier adaptive immune response. One might speculate that increased killing of *S. suis cps*2 552 in blood of last-born piglets in the 6th week of life in this study is a result of this early adaptive immune response. However, there are important critical and unresolved points to this interpretation: As mentioned above, B1-like cells are an important source of IgM binding to *S. suis* after weaning [45]. B1 cells exhibit innate-like functions [46, 48]. It is currently unknown if maternal immunity exhibits an influence on the activation of B cells secreting IgM and IgA binding to *S. suis* after weaning. Of note, piglets of non-vaccinated and vaccinated sows showed a comparable increase in levels of IgM and IgA binding to *S. suis cps*1 after weaning in a *S. suis cps*1 infected herd [28].

Based on the result of the LMM analysis, the trait body weight has a positive and time independent effect on the transformed survival factor of *S. suis cps*2 (body weight as measured at 2-, 6- and 10-weeks of age). Further studies are needed to clarify if this association is true for piglets in general. However, this finding agrees with our experience that well-developed piglets often get *S. suis* septicemia and meningitis. One might speculate that this association is due to a genetic linkage between weight gain and susceptibility to *S. suis* bacteremia. Of note, blood leukocytes show differences in the generation of cytokines such as TNFα upon exposure to bacterial pathogens in dependence of the pig breed [49]. Notably, the low divergence of survival factors observed within numerous litters and the prominent differences between some litters (Fig. 6) might also be related to heritability.

## Conclusions

This field study demonstrates that serum IgG and serum IgA levels in 2-week-old suckling piglets are mainly determined by respective levels in colostrum in the investigated herd with autogenous *S. suis* bacterin vaccination pre-farrowing. Importantly, it reveals for the first time a statistically significant effect of serum IgA binding to *S. suis cps*2 strain on survival of this strain in porcine blood at 2, 6 and 10 weeks of age. In contrast, specific total IgG levels do not show such an effect. Specific IgA levels might increase after 2 weeks of age whereas specific IgG levels are still decreasing. This study further supports the concept that IgG-independent mechanisms play a crucial role in controlling *S. suis* bacteremia during the critical phase of piglet rearing.

## Supplementary Information

Below is the link to the electronic supplementary material.

Supplementary Material 1

Supplementary Material 2

Supplementary Material 3

Supplementary Material 4

Supplementary Material 5

Supplementary Material 6

Supplementary Material 7

Supplementary Material 8

Supplementary Material 9

## Data Availability

Data is available under [doi.org/10.5281/zenodo.16994131](https:/doi.org/10.5281/zenodo.16994131) . Non-commercial material is available from the corresponding author on reasonable request.
