# Supplementary material for: Serum IgA and bactericidal immunity against Streptococcus suis serotype 2 is increasing between 2 and 6 weeks of age in a farm with autogenous bacterin vaccination pre-farrowing, while specific maternal IgG is decreasing
Source: Porcine Health Manag. 2026 Jan 14;12:5. doi: 10.1186/s40813-025-00485-y (PMC12896002; doi:10.1186/s40813-025-00485-y)
Supplement: Supplementary file 1 — Supplementary Material 1 [file 40813_2025_485_MOESM1_ESM.pdf]

Supplementary Material 1

Table S1: Letter designation, parity and number of piglets of the investigated litters of dams

Gilts			Middle aged sows			Old sows		
Letter (litter)	Parity	Live born piglets	Letter (litter)	Parity	Live born piglets	Letter (litter)	Parity	Live born piglets
A	1	16	C	4	18	B	6	17
E	1	15	D	2	19	F	8	15
G	1	16	I	3	18	H	6	17
K	1	18	J	3	19	L	7	20
M	1	18	O	3	16	N	5	28
Q	1	16	P	2	13	R	7	21
S	1	17	U	3	18	T	7	20
W	1	13	V	3	17	X	5	16
