# Supplementary material for: Serum IgA and bactericidal immunity against Streptococcus suis serotype 2 is increasing between 2 and 6 weeks of age in a farm with autogenous bacterin vaccination pre-farrowing, while specific maternal IgG is decreasing
Source: Porcine Health Manag. 2026 Jan 14;12:5. doi: 10.1186/s40813-025-00485-y (PMC12896002; doi:10.1186/s40813-025-00485-y)
Supplement: Supplementary file 3 — Supplementary Material 3 [file 40813_2025_485_MOESM3_ESM.pdf]

Supplementary Material 3:

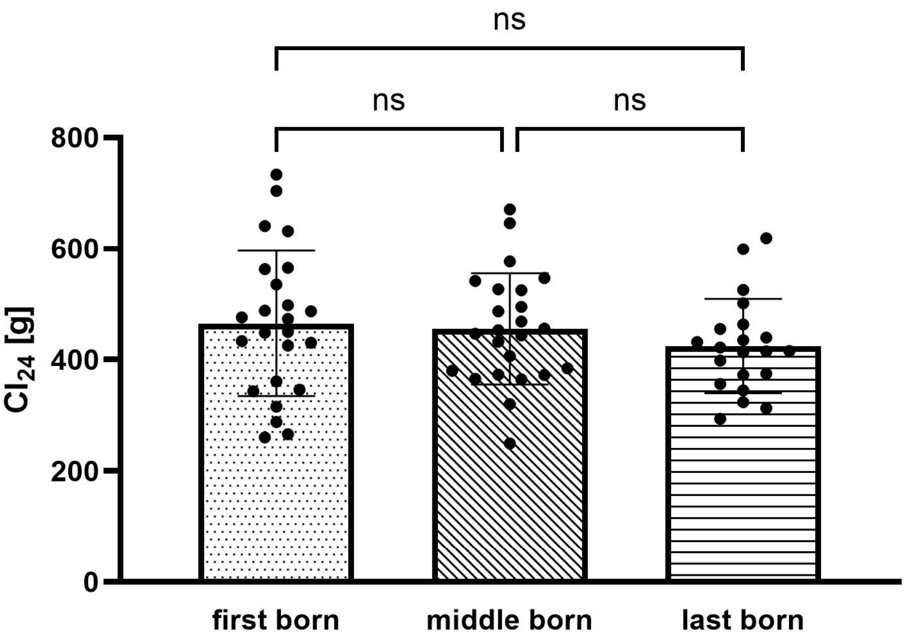

	first born	middle born	last born
mean:	465.4	455.6	424.6
SD:	130.8	100.0	84.90
n:	24	24	21

Supplementary Figure 3: Comparison of the amount of colostrum taken up in the first 24 h (CI_{24}) between first-, middle- and last-born piglets of the investigated litters. Statistical analysis was conducted with the one-way ANOVA with Tukey’s test.
