# Supplementary material for: Serum IgA and bactericidal immunity against Streptococcus suis serotype 2 is increasing between 2 and 6 weeks of age in a farm with autogenous bacterin vaccination pre-farrowing, while specific maternal IgG is decreasing
Source: Porcine Health Manag. 2026 Jan 14;12:5. doi: 10.1186/s40813-025-00485-y (PMC12896002; doi:10.1186/s40813-025-00485-y)
Supplement: Supplementary file 4 — Supplementary Material 4 [file 40813_2025_485_MOESM4_ESM.pdf]

Supplementary Material 4:

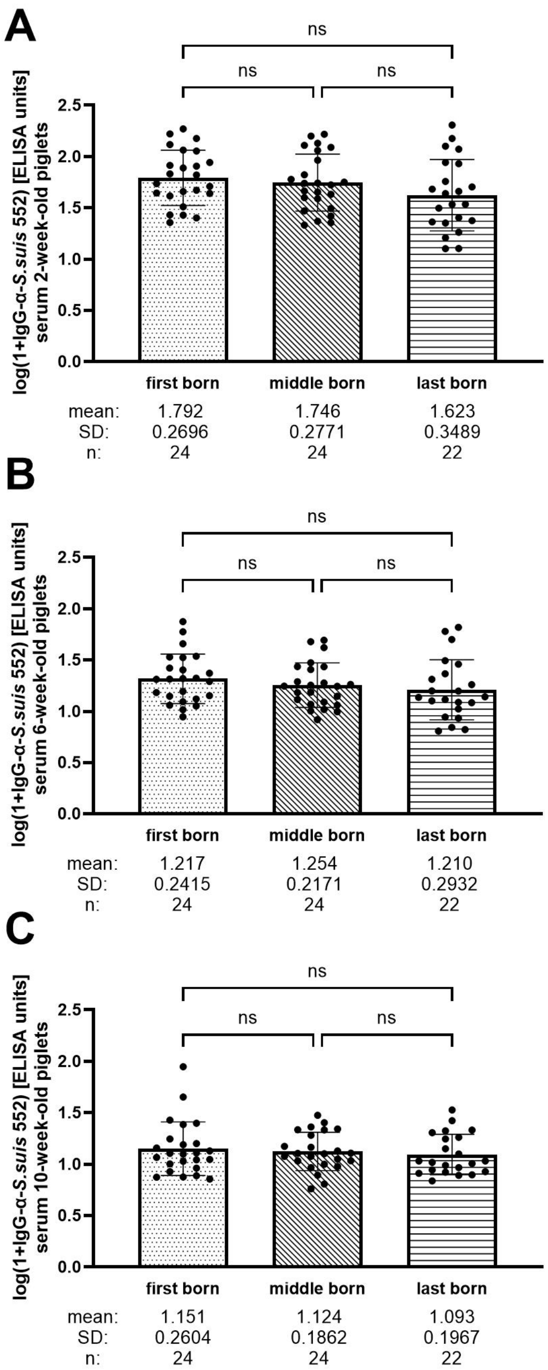

Supplementary Figure 4: Levels of IgG binding to *S. suis cps*2 strain 552 in serum samples drawn from 2 (A), 6 (B) and 8 (C) week old piglets. In each litter a first-, middle- and last-born piglet was investigated except for 2 last born piglets that died early. Statistical analysis was conducted with the one-way ANOVA with Tukey’s test.
