# Supplementary material for: Serum IgA and bactericidal immunity against Streptococcus suis serotype 2 is increasing between 2 and 6 weeks of age in a farm with autogenous bacterin vaccination pre-farrowing, while specific maternal IgG is decreasing
Source: Porcine Health Manag. 2026 Jan 14;12:5. doi: 10.1186/s40813-025-00485-y (PMC12896002; doi:10.1186/s40813-025-00485-y)
Supplement: Supplementary file 5 — Supplementary Material 5 [file 40813_2025_485_MOESM5_ESM.pdf]

Supplementary Material 5:

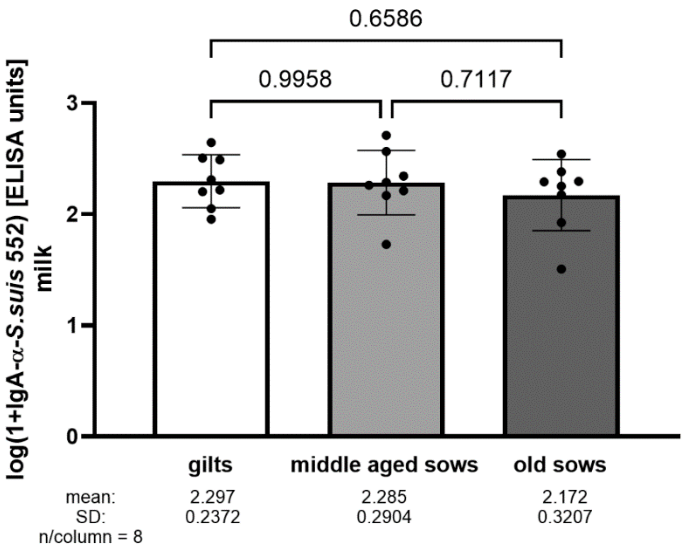

Supplementary Figure 5: Levels of IgA binding to *S. suis cps*2 strain 552 in milk collected from vaccinated dams one day after weaning. Vaccination with a multivalent S. suis vaccine was conducted as shown in Fig. 1. A
