# Supplementary material for: Serum IgA and bactericidal immunity against Streptococcus suis serotype 2 is increasing between 2 and 6 weeks of age in a farm with autogenous bacterin vaccination pre-farrowing, while specific maternal IgG is decreasing
Source: Porcine Health Manag. 2026 Jan 14;12:5. doi: 10.1186/s40813-025-00485-y (PMC12896002; doi:10.1186/s40813-025-00485-y)
Supplement: Supplementary file 6 — Supplementary Material 6 [file 40813_2025_485_MOESM6_ESM.pdf]

Supplementary Material 6:

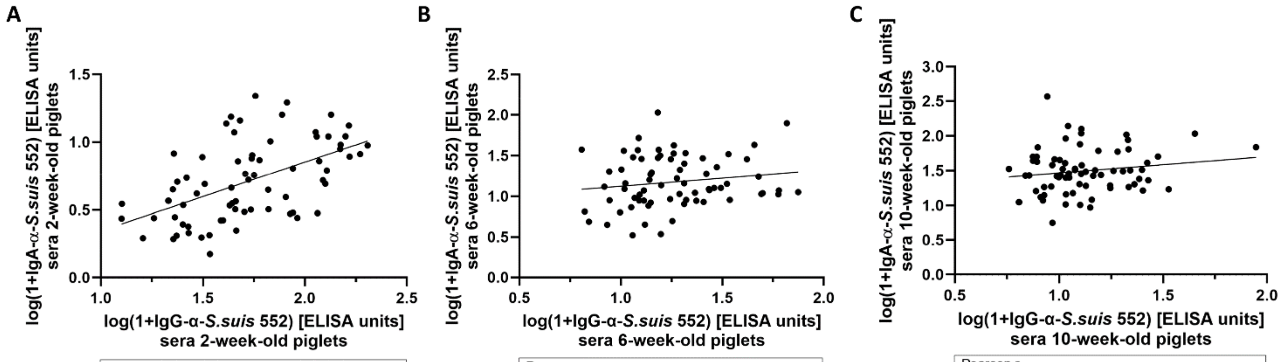

Pearson r	
r	0.5320
95% confidence interval	0.3395 to 0.6818
R squared	0.2831
P value (two-tailed)	<0.0001 (****)
Significant? (alpha = 0.05)	Yes
Number of XY Pairs	70

Pearson r	
r	0.1575
95% confidence interval	-0.08050 to 0.3784
R squared	0.02479
P value (two-tailed)	0.1930 (ns)
Significant? (alpha = 0.05)	No
Number of XY Pairs	70

Pearson r	
r	0.1578
95% confidence interval	-0.08013 to 0.3787
R squared	0.02491
P value (two-tailed)	0.1920 (ns)
Significant? (alpha = 0.05)	No
Number of XY Pairs	70

Supplementary Figure 6: Pearson correlation analysis of levels of specific IgA and IgG binding to *S. suis cps*2 552 at 2 (A), 6 (B) and 10 (C) weeks of age.
