# Supplementary material for: Serum IgA and bactericidal immunity against Streptococcus suis serotype 2 is increasing between 2 and 6 weeks of age in a farm with autogenous bacterin vaccination pre-farrowing, while specific maternal IgG is decreasing
Source: Porcine Health Manag. 2026 Jan 14;12:5. doi: 10.1186/s40813-025-00485-y (PMC12896002; doi:10.1186/s40813-025-00485-y)
Supplement: Supplementary file 7 — Supplementary Material 7 [file 40813_2025_485_MOESM7_ESM.pdf]

Supplementary Material 7:

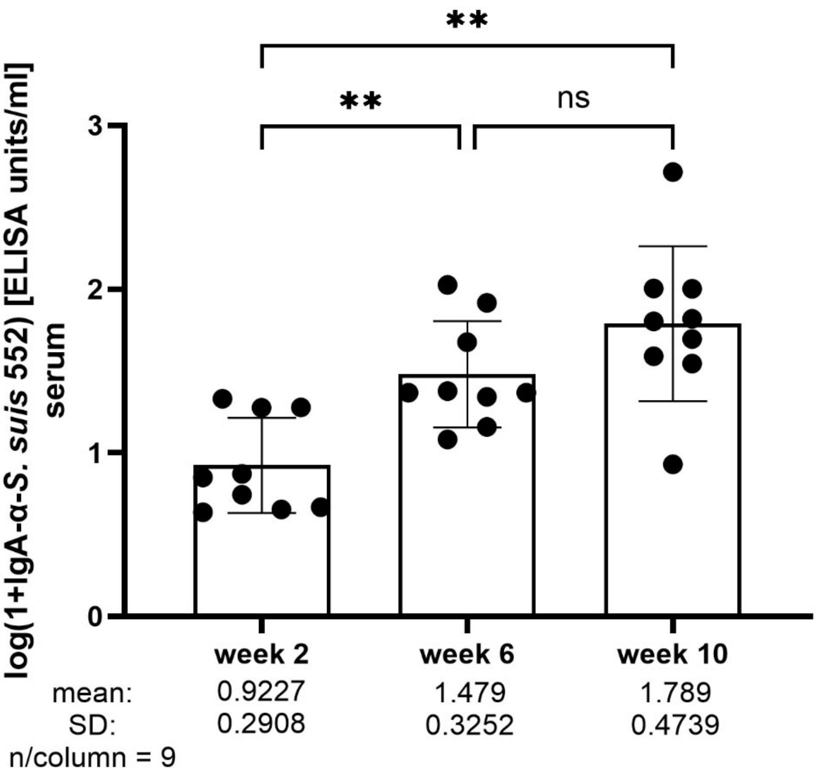

Supplementary Figure 7: Levels of specific serum IgA antibodies binding to surface-associated antigens of *S. suis* cps2 strain 552 in 2-, 6- and 10-week-old piglets detected via an ELISA using a monoclonal anti IgA antibody against IgA. The diagram shows the results of the middle-born piglets of different dams.
