# Supplementary material for: Serum IgA and bactericidal immunity against Streptococcus suis serotype 2 is increasing between 2 and 6 weeks of age in a farm with autogenous bacterin vaccination pre-farrowing, while specific maternal IgG is decreasing
Source: Porcine Health Manag. 2026 Jan 14;12:5. doi: 10.1186/s40813-025-00485-y (PMC12896002; doi:10.1186/s40813-025-00485-y)
Supplement: Supplementary file 8 — Supplementary Material 8 [file 40813_2025_485_MOESM8_ESM.pdf]

Supplementary material 8

Results of linear mixed-effects models for all investigated parameters on survival of *S. suis cps*2 strain 552 in blood of piglets drawn at 2, 6 and 10 weeks of age

Table of Contents

1. Strain S. suis 552 2
    1.1 Litter Order 2
    1.2 Dam Parity 3
    1.3 Piglet Birth Weight (Day0) [kg] 4
    1.4 Piglet Weight after 24 Hours (Day1) [kg] 4
    1.5 Piglet Weight in 2nd to 10th Postnatal Week (Weight) [kg] 5
    1.6 Colostrum Intake During the First 24 Hours (CI24) [kg] 6
    1.7 IgG Colostrum [log ELISA Units] 7
    1.8 IgG Serum [log ELISA Units] 8
    1.9 IgA Colostrum [log ELISA Units] 8
    1.10 IgA Serum [log ELISA Units] 9

1. Strain S. suis 552

<u>M1 (Default Model):</u>

Random effects:

Group	n	Parameter	Variance	Std. Dev.
Piglet:Dam	70	(Intercept)	0.0000	0.0000
		Time	0.0000	0.0000
Dam	24	(Intercept)	0.4101	0.6404
		Time	0.0114	0.1070
Residual			0.8121	0.9011

Fixed effects:

Parameter	Estimate β	95% CI	Std. Error	P value
(Intercept)	-1.0608	[-1.4234, -0.6975]	0.1846	**<0.001**
Time	-0.1585	[-0.2154, -0.1012]	0.0290	**<0.001**

1.1 Litter Order

<u>M2 (Main Effect Model):</u>

Random effects:

Group	n	Parameter	Variance	Std. Dev.
Piglet:Dam	70	(Intercept)	0.0000	0.0000
		Time	0.0000	0.0000
Dam	24	(Intercept)	0.4018	0.6338
		Time	0.0114	0.1068
Residual			0.8161	0.9034

Fixed effects:

Parameter	Estimate β	95% CI	Std. Error	P value
(Intercept)	-1.0641	[-1.4249, -0.7046]	0.1840	**<0.001**
Time	-0.1585	[-0.2157, -0.1018]	0.0290	**<0.001**
Litter Order (L)*	-0.1200	[-0.3358, 0.0956]	0.1093	0.274
Litter Order (Q)*	-0.0266	[-0.2367, 0.1852]	0.1074	0.805

<u>M3 (Interaction Model):</u>

Random effects:

Group	n	Parameter	Variance	Std. Dev.
Piglet:Dam	70	(Intercept)	0.0000	0.0000
		Time	0.0000	0.0000
Dam	24	(Intercept)	0.4249	0.6518
		Time	0.0120	0.1097
Residual			0.8096	0.8998

Fixed effects:

Parameter	Estimate β	95% CI	Std. Error	P value
(Intercept)	-1.0546	[-1.4204, -0.6890]	0.1863	**<0.001**
Time	-0.1601	[-0.2178, -0.1023]	0.0294	**<0.001**
Litter Order (L)*	0.1090	[-0.3358, 0.5551]	0.2278	0.633
Litter Order (Q)*	0.2078	[-0.2311, 0.6468]	0.2238	0.355
Time:Litter Order (L)*	-0.0382	[-0.1038, 0.0270]	0.0334	0.254
Time:Litter Order (Q)*	-0.0391	[-0.1031, 0.0250]	0.0328	0.235

Model Comparison:

Model	npar	Marginal R^2	Conditional R^2	Log-Likelihood	P value
M1	9	0.2070	0.3755	-293.51	M2 vs M1: 0.5322
M2	11	0.2095	0.3763	-292.88	M3 vs M1: 0.4166
M3	13	0.2163	0.3872	-291.55	M3 vs M2: 0.2644

1.2 Dam Parity

M2 (Main Effect Model):

Random effects:

Group	n	Parameter	Variance	Std. Dev.
Piglet:Dam	70	(Intercept)<br>Time	0.0000<br>0.0000	0.0000<br>0.0000
Dam	24	(Intercept)<br>Time	0.4268<br>0.0114	0.6533<br>0.1070
Residual			0.8125	0.9014

Fixed effects:

Parameter	Estimate β	95% CI	Std. Error	P value
(Intercept)	-1.0607	[-1.4261, -0.6932]	0.1866	**<0.001**
Time	-0.1585	[-0.2156, -0.1017]	0.0290	**<0.001**
Dam Parity (L)*	-0.0499	[-0.3715, 0.2704]	0.1586	0.756
Dam Parity (Q)*	-0.0556	[-0.3746, 0.2644]	0.1592	0.731

M3 (Interaction Model):

Random effects:

Group	n	Parameter	Variance	Std. Dev.
Piglet:Dam	70	(Intercept)<br>Time	0.0000<br>0.0000	0.0000<br>0.0000
Dam	24	(Intercept)<br>Time	0.4804<br>0.0131	0.6931<br>0.1146
Residual			0.8120	0.9011

Fixed effects:

Parameter	Estimate β	95% CI	Std. Error	P value
(Intercept)	-1.0586	[-1.4364, -0.6806]	0.1925	**<0.001**
Time	-0.1588	[-0.2180, -0.0994]	0.0302	**<0.001**
Dam Parity (L)*	0.0893	[-0.5629, 0.7423]	0.3327	0.791
Dam Parity (Q)*	-0.1397	[-0.7962, 0.5118]	0.3340	0.680
Time:Dam Parity (L)*	-0.0249	[-0.1276, 0.0772]	0.0522	0.639
Time:Dam Parity (Q)*	0.0150	[-0.0878, 0.1177]	0.0524	0.777

Model Comparison:

Model	npar	Marginal R^2	Conditional R^2	Log-Likelihood	P value
M1	9	0.2070	0.3755	-293.51	M2 vs M1: 0.8780
M2	11	0.2058	0.3832	-293.38	M3 vs M1: 0.9625
M3	13	0.2049	0.3934	-293.21	M3 vs M2: 0.8415

*Since Litter Order and Dam Parity are ordinal variables with three levels, two orthogonal polynomial contrasts (one fewer than the number of levels) are fitted to the levels. While the first contrast (L) is linear, the second contrast (Q) is quadratic.

1.3 Piglet Birth Weight (Day0) [kg]

M2 (Main Effect Model):

Random effects:

Group	n	Parameter	Variance	Std. Dev.
Piglet:Dam	69	(Intercept)<br>Time	0.0000<br>0.0000	0.0001<br>0.0000
Dam	24	(Intercept)<br>Time	0.4493<br>0.0116	0.6703<br>0.1079
Residual			0.7868	0.8870

Fixed effects:

Parameter	Estimate β	95% CI	Std. Error	P value
(Intercept)	-0.6755	[-1.5813, 0.2313]	0.4519	0.138
Time	-0.1575	[-0.2143, -0.1007]	0.0291	**<0.001**
Day0	-0.2701	[-0.8653, 0.3251]	0.2953	0.363

M3 (Interaction Model):

Random effects:

Group	n	Parameter	Variance	Std. Dev.
Piglet:Dam	69	(Intercept)<br>Time	0.0000<br>0.0000	0.0005<br>0.0001
Dam	24	(Intercept)<br>Time	0.4491<br>0.0118	0.6701<br>0.1085
Residual			0.7900	0.8888

Fixed effects:

Parameter	Estimate β	95% CI	Std. Error	P value
(Intercept)	-1.0033	[-2.7865, 0.7755]	0.8939	0.265
Time	-0.1020	[-0.3702, 0.1664]	0.1345	0.450
Day0	-0.0341	[-1.2912, 1.2198]	0.6287	0.957
Time:Day0	-0.0400	[-0.2279, 0.1489]	0.0945	0.673

Model Comparison:

Model	npar	Marginal R^2	Conditional R^2	Log-Likelihood	P value
M1	9	0.2104	0.3746	-286.16	M2 vs M1: 0.3684
M2	10	0.2129	0.3810	-285.76	M3 vs M1: 0.6062
M3	11	0.2133	0.3816	-285.66	M3 vs M2: 0.6611

1.4 Piglet Weight after 24 Hours (Day1) [kg]

M2 (Main Effect Model):

Random effects:

Group	n	Parameter	Variance	Std. Dev.
Piglet:Dam	69	(Intercept)<br>Time	0.0000<br>0.0000	0.0001<br>0.0000
Dam	24	(Intercept)<br>Time	0.4470<br>0.0117	0.6686<br>0.1080
Residual			0.7836	0.8852

Fixed effects:

Parameter	Estimate β	95% CI	Std. Error	P value
(Intercept)	-0.5635	[-1.4289, 0.3059]	0.4372	0.200
Time	-0.1575	[-0.2149, -0.1008]	0.0291	**<0.001**
Day1	-0.3288	[-0.8621, 0.2031]	0.2663	0.220

M3 (Interaction Model):

Random effects:

Group	n	Parameter	Variance	Std. Dev.
Piglet:Dam	69	(Intercept)<br>Time	0.0000<br>0.0000	0.0001<br>0.0000
Dam	24	(Intercept)<br>Time	0.4532<br>0.0119	0.6732<br>0.1093
Residual			0.7867	0.8869

Fixed effects:

Parameter	Estimate β	95% CI	Std. Error	P value
(Intercept)	-0.7396	[-2.4590, 0.9755]	0.8601	0.392
Time	-0.1277	[-0.3848, 0.1294]	0.1292	0.325
Day1	-0.2098	[-1.3402, 0.9239]	0.5663	0.712
Time:Day1	-0.0201	[-0.1900, 0.1479]	0.0850	0.813

Model Comparison:

Model	npar	Marginal R^2	Conditional R^2	Log-Likelihood	P value
M1	9	0.2104	0.3746	-286.16	M2 vs M1: 0.2211
M2	10	0.2156	0.3840	-285.41	M3 vs M1: 0.4591
M3	11	0.2151	0.3848	-285.38	M3 vs M2: 0.8064

1.5 Piglet Weight in 2nd to 10th Postnatal Week (Weight) [kg]

M2 (Main Effect Model):

Random effects:

Group	n	Parameter	Variance	Std. Dev.
Piglet:Dam	70	(Intercept)<br>Time	0.0000<br>0.0000	0.0000<br>0.0001
Dam	24	(Intercept)<br>Time	0.4062<br>0.0111	0.6373<br>0.1054
Residual			0.8010	0.8950

Fixed effects:

Parameter	Estimate β	95% CI	Std. Error	P value
(Intercept)	-0.9623	[-1.3304, -0.5929]	0.1884	**<0.001**
Time	-0.2647	[-0.3716, -0.1583]	0.0542	**<0.001**
Weight	0.0425	[0.0062, 0.0786]	0.0184	**0.0219**

M3 (Interaction Model):

Random effects:

Group	n	Parameter	Variance	Std. Dev.
Piglet:Dam	70	(Intercept)<br>Time	0.0000<br>0.0000	0.0003<br>0.0001
Dam	24	(Intercept)<br>Time	0.4186<br>0.0112	0.6470<br>0.1058
Residual			0.7981	0.8934

Fixed effects:

Parameter	Estimate β	95% CI	Std. Error	P value
(Intercept)	-0.7177	[-1.4459, 0.0107]	0.3687	0.0535
Time	-0.2658	[-0.3727, -0.1588]	0.0542	**<0.001**
Weight	-0.0190	[-0.1804, 0.1426]	0.0812	0.8152
Time:Weight	0.0053	[-0.0082, 0.0187]	0.0068	0.4395

Model Comparison:

Model	npar	Marginal R^2	Conditional R^2	Log-Likelihood	P value
M1	9	0.2070	0.3755	-293.51	M2 vs M1: **0.0207**
M2	10	0.2258	0.3827	-290.83	M3 vs M1: 0.0521
M3	11	0.2252	0.3879	-290.55	M3 vs M2: 0.4573

1.6 Colostrum Intake During the First 24 Hours (CI24) [kg]

M2 (Main Effect Model):

Random effects:

Group	n	Parameter	Variance	Std. Dev.
Piglet:Dam	69	(Intercept)<br>Time	0.0000<br>0.0000	0.0001<br>0.0000
Dam	24	(Intercept)<br>Time	0.4201<br>0.0117	0.6482<br>0.1081
Residual			0.7838	0.8853

Fixed effects:

Parameter	Estimate β	95% CI	Std. Error	P value
(Intercept)	-0.6172	[-1.3089, 0.0782]	0.3482	0.0788
Time	-0.1575	[-0.2143, -0.1009]	0.0291	**<0.001**
CI24	-0.9667	[-2.2723, 0.3369]	0.6570	0.1436

M3 (Interaction Model):

Random effects:

Group	n	Parameter	Variance	Std. Dev.
Piglet:Dam	69	(Intercept)<br>Time	0.0000<br>0.0000	0.0009<br>0.0001
Dam	24	(Intercept)<br>Time	0.4232<br>0.0117	0.6505<br>0.1083
Residual			0.7877	0.8875

Fixed effects:

Parameter	Estimate β	95% CI	Std. Error	P value
(Intercept)	-0.4438	[-1.7377, 0.8433]	0.6508	0.497
Time	-0.1871	[-0.3817, 0.0071]	0.0978	0.058
CI24	-1.3533	[-4.0996, 1.4288]	1.3903	0.332
Time:CI24	0.0659	[-0.3470, 0.4801]	0.2080	0.752

Model Comparison:

Model	npar	Marginal R^2	Conditional R^2	Log-Likelihood	P value
M1	9	0.2104	0.3746	-286.16	M2 vs M1: 0.1412
M2	10	0.2177	0.3832	-285.08	M3 vs M1: 0.3210
M3	11	0.2171	0.3821	-285.03	M3 vs M2: 0.7421

1.7 IgG Colostrum [log ELISA Units]

M2 (Main Effect Model):

Random effects:

Group	n	Parameter	Variance	Std. Dev.
Piglet:Dam	70	(Intercept)<br>Time	0.0000<br>0.0000	0.0008<br>0.0001
Dam	24	(Intercept)<br>Time	0.4554<br>0.0115	0.6748<br>0.1070
Residual			0.8118	0.9010

Fixed effects:

Parameter	Estimate β	95% CI	Std. Error	P value
(Intercept)	-0.1435	[-1.7807, 1.4850]	0.8174	0.862
Time	-0.1585	[-0.2151, -0.1021]	0.0290	**<0.001**
IgG Colostrum	-0.1545	[-0.4226, 0.1152]	0.1340	0.261

M3 (Interaction Model):

Random effects:

Group	n	Parameter	Variance	Std. Dev.
Piglet:Dam	70	(Intercept)<br>Time	0.0000<br>0.0000	0.0001<br>0.0000
Dam	24	(Intercept)<br>Time	0.4320<br>0.0108	0.6572<br>0.1038
Residual			0.8113	0.9007

Fixed effects:

Parameter	Estimate β	95% CI	Std. Error	P value
(Intercept)	-2.2002	[-5.5580, 1.1452]	1.7019	0.210
Time	0.1966	[-0.3108, 0.7064]	0.2595	0.457
IgG Colostrum	0.1920	[-0.3676, 0.7543]	0.2851	0.508
Time:IgG Colostrum	-0.0598	[-0.1450, 0.0251]	0.0435	0.183

Model Comparison:

Model	npar	Marginal R^2	Conditional R^2	Log-Likelihood	P value
M1	9	0.2070	0.3755	-293.51	M2 vs M1: 0.2540
M2	10	0.2140	0.3788	-292.86	M3 vs M1: 0.1935
M3	11	0.2257	0.3838	-291.87	M3 vs M2: 0.1590

1.8 IgG Serum [log ELISA Units]

M2 (Main Effect Model):

Random effects:

Group	n	Parameter	Variance	Std. Dev.
Piglet:Dam	70	(Intercept)<br>Time	0.0000<br>0.0000	0.0000<br>0.0000
Dam	24	(Intercept)<br>Time	0.4139<br>0.0113	0.6434<br>0.1061
Residual			0.8123	0.9013

Fixed effects:

Parameter	Estimate β	95% CI	Std. Error	P value
(Intercept)	-1.2812	[-2.4012, -0.1626]	0.5551	**0.0235**
Time	-0.1494	[-0.2211, -0.0779]	0.0361	**<0.001**
IgG Serum	0.0526	[-0.1992, 0.3053]	0.1248	0.6747

M3 (Interaction Model):

Random effects:

Group	n	Parameter	Variance	Std. Dev.
Piglet:Dam	70	(Intercept)<br>Time	0.0000<br>0.0000	0.0003<br>0.0001
Dam	24	(Intercept)<br>Time	0.4280<br>0.0116	0.6542<br>0.1077
Residual			0.8120	0.9011

Fixed effects:

Parameter	Estimate β	95% CI	Std. Error	P value
(Intercept)	-1.6840	[-3.1476, -0.2181]	0.7373	**0.0244**
Time	-0.0656	[-0.2756, 0.1440]	0.1064	0.5380
IgG Serum	0.1732	[-0.2062, 0.5524]	0.1911	0.3665
Time:IgG Serum	-0.0281	[-0.0943, 0.0383]	0.0335	0.4027

Model Comparison:

Model	npar	Marginal R^2	Conditional R^2	Log-Likelihood	P value
M1	9	0.2070	0.3755	-293.51	M2 vs M1: 0.6981
M2	10	0.2065	0.3788	-293.43	M3 vs M1: 0.6536
M3	11	0.2083	0.3822	-293.08	M3 vs M2: 0.4027

1.9 IgA Colostrum [log ELISA Units]

M2 (Main Effect Model):

Random effects:

Group	n	Parameter	Variance	Std. Dev.
Piglet:Dam	70	(Intercept)<br>Time	0.0000<br>0.0000	0.0002<br>0.0000
Dam	24	(Intercept)<br>Time	0.3968<br>0.0115	0.6299<br>0.1070
Residual			0.8116	0.9009

Fixed effects:

Parameter	Estimate β	95% CI	Std. Error	P value
(Intercept)	0.0932	[-1.8245, 2.0179]	0.9536	0.923
Time	-0.1585	[-0.2153, -0.1014]	0.0290	**<0.001**
IgA Colostrum	-0.1906	[-0.5023, 0.1216]	0.1545	0.231

M3 (Interaction Model):

Random effects:

Group	n	Parameter	Variance	Std. Dev.
Piglet:Dam	70	(Intercept)<br>Time	0.0000<br>0.0000	0.0001<br>0.0000
Dam	24	(Intercept)<br>Time	0.4241<br>0.0123	0.6512<br>0.1110
Residual			0.8115	0.9008

Fixed effects:

Parameter	Estimate β	95% CI	Std. Error	P value
(Intercept)	0.5681	[-3.3676, 4.5049]	2.0011	0.779
Time	-0.2442	[-0.8711, 0.3804]	0.3186	0.452
IgA Colostrum	-0.2689	[-0.9158, 0.3772]	0.3291	0.423
Time:IgA Colostrum	0.0141	[-0.0885, 0.1173]	0.0524	0.790

Model Comparison:

Model	npar	Marginal R^2	Conditional R^2	Log-Likelihood	P value
M1	9	0.2070	0.3755	-293.51	M2 vs M1: 0.2063
M2	10	0.2146	0.3801	-292.71	M3 vs M1: 0.4324
M3	11	0.2132	0.3846	-292.67	M3 vs M2: 0.7782

1.10 IgA Serum [log ELISA Units]

M2 (Main Effect Model):

Random effects:

Group	n	Parameter	Variance	Std. Dev.
Piglet:Dam	70	(Intercept)<br>Time	0.0000<br>0.0000	0.0000<br>0.0000
Dam	24	(Intercept)<br>Time	0.4179<br>0.0108	0.6464<br>0.1037
Residual			0.8072	0.8985

Fixed effects:

Parameter	Estimate β	95% CI	Std. Error	P value
(Intercept)	-0.8062	[-1.2518, -0.3647]	0.2251	**<0.001**
Time	-0.1121	[-0.1853, -0.0395]	0.0368	**0.0036**
IgA Serum	-0.2052	[-0.4113, -0.0022]	0.1032	**0.0484**

M3 (Interaction Model):

Random effects:

Group	n	Parameter	Variance	Std. Dev.
Piglet:Dam	70	(Intercept)<br>Time	0.0000<br>0.0000	0.0002<br>0.0000
Dam	24	(Intercept)<br>Time	0.4691<br>0.0126	0.6849<br>0.1123
Residual			0.7808	0.8836

Fixed effects:

Parameter	Estimate β	95% CI	Std. Error	P value
(Intercept)	-0.1626	[-0.9240, 0.5918]	0.3821	0.6713
Time	-0.2436	[-0.3875, -0.0993]	0.0724	**<0.001**
IgA Serum	-0.5023	[-0.8513, -0.1531]	0.1756	**0.0048**
Time:IgA Serum	0.0510	[0.0029, 0.0990]	0.0242	**0.0361**

Model Comparison:

Model	npar	Marginal R^2	Conditional R^2	Log-Likelihood	P value
M1	9	0.2070	0.3755	-293.51	M2 vs M1: **0.0470**
M2	10	0.2233	0.3788	-291.54	M3 vs M1: **0.0169**
M3	11	0.2349	0.4080	-289.43	M3 vs M2: **0.0400**
