# Supplementary material for: Serum IgA and bactericidal immunity against Streptococcus suis serotype 2 is increasing between 2 and 6 weeks of age in a farm with autogenous bacterin vaccination pre-farrowing, while specific maternal IgG is decreasing
Source: Porcine Health Manag. 2026 Jan 14;12:5. doi: 10.1186/s40813-025-00485-y (PMC12896002; doi:10.1186/s40813-025-00485-y)
Supplement: Supplementary file 9 — Supplementary Material 9 [file 40813_2025_485_MOESM9_ESM.pdf]

Supplementary Material 9:

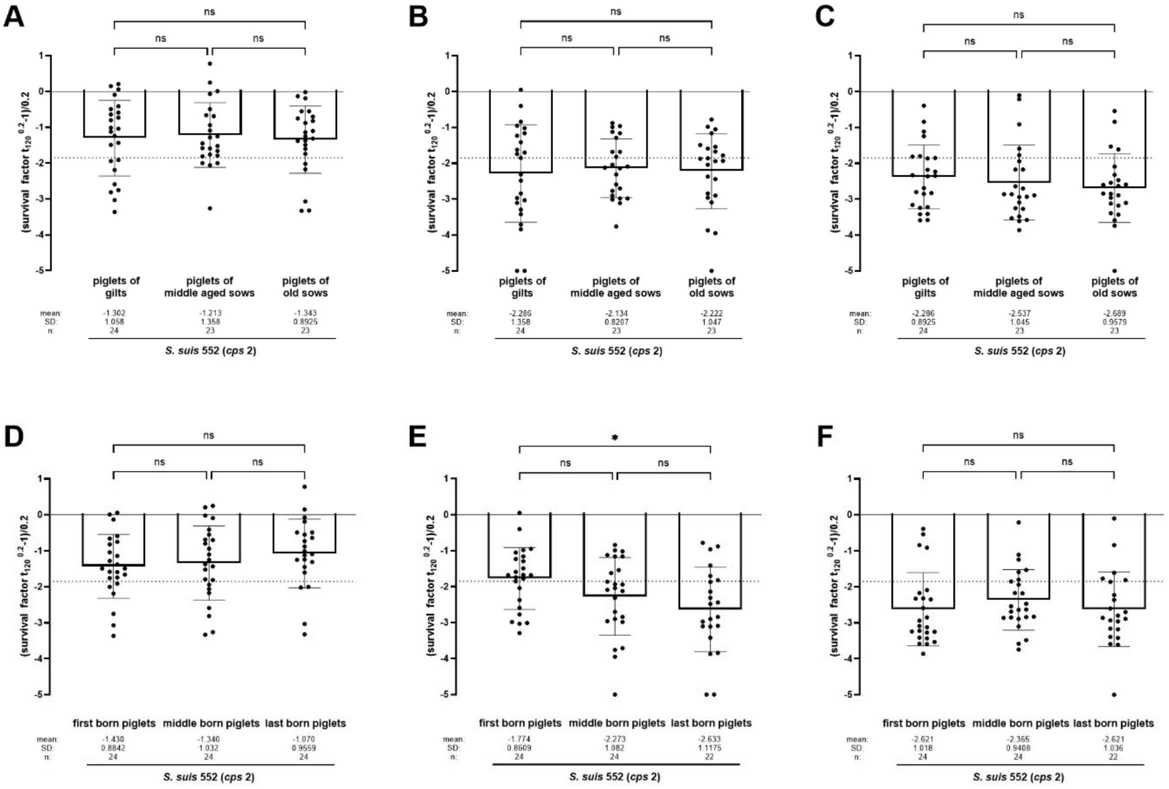

Supplementary Figure 9: Comparison of Box-Cox-transformed survival factors of *S. suis cps*2 552 in blood of the indicated groups of piglets at an age of 2 (A, D), 6 (B, E) and 10 weeks (C, F). (A to C) Piglets of gilts, middle-aged and old sows are compared. Each litter is represented with three animals. (D to F) Comparison of first, middle and last-born piglets. Each investigated litter is represented with one piglet in each group.
